# Exploring the potential effects of forest urbanization on the interplay between small mammal communities and their gut microbiota

**DOI:** 10.1186/s42523-024-00301-y

**Published:** 2024-03-25

**Authors:** Marie Bouilloud, Maxime Galan, Julien Pradel, Anne Loiseau, Julien Ferrero, Romain Gallet, Benjamin Roche, Nathalie Charbonnel

**Affiliations:** 1 CBGP, IRD, CIRAD, INRAE, Institut Agro, Univ Montpellier, Montpellier, France; 2 MIVEGEC, IRD, CNRS, Univ Montpellier, Montpellier, France; 3 Centre de Biologie pour la Gestion des Populations, 750 Avenue Agropolis, 34988 Montferrier sur Lez, France

**Keywords:** Community, Dysbiosis, Bacterial gut microbiota, Rodents, Urban ecology, Wildlife

## Abstract

Urbanization significantly impacts wild populations, favoring urban dweller species over those that are unable to adapt to rapid changes. These differential adaptative abilities could be mediated by the microbiome, which may modulate the host phenotype rapidly through a high degree of flexibility. Conversely, under anthropic perturbations, the microbiota of some species could be disrupted, resulting in dysbiosis and negative impacts on host fitness. The links between the impact of urbanization on host communities and their gut microbiota (GM) have only been scarcely explored. In this study, we tested the hypothesis that the bacterial composition of the GM could play a role in host adaptation to urban environments. We described the GM of several species of small terrestrial mammals sampled in forested areas along a gradient of urbanization, using a 16S metabarcoding approach. We tested whether urbanization led to changes in small mammal communities and in their GM, considering the presence and abundance of bacterial taxa and their putative functions. This enabled to decipher the processes underlying these changes. We found potential impacts of urbanization on small mammal communities and their GM. The urban dweller species had a lower bacterial taxonomic diversity but a higher functional diversity and a different composition compared to urban adapter species. Their GM assembly was mostly governed by stochastic effects, potentially indicating dysbiosis. Selection processes and an overabundance of functions were detected that could be associated with adaptation to urban environments despite dysbiosis. In urban adapter species, the GM functional diversity and composition remained relatively stable along the urbanization gradient. This observation can be explained by functional redundancy, where certain taxa express the same function. This could favor the adaptation of urban adapter species in various environments, including urban settings. We can therefore assume that there are feedbacks between the gut microbiota and host species within communities, enabling rapid adaptation.

**Supplementary Information:**

The online version contains supplementary material available at 10.1186/s42523-024-00301-y.

## Introduction

Urbanization, the process of making an area more urban through higher human population presence and occupancy within cities [42, 100, 103], is a major driver of global land use change. It is associated with increased rates of habitat loss or fragmentation, often coupled with diversity loss and species extinction (e.g. [21, 65]). Global assessments report that urban expansion can be responsible for a 50% loss of local species richness [56]. Urbanization may induce rapid and abrupt changes that hinder the adaptation of species reliant on specific natural environments [73], ultimately leading to the extinction of entire populations [88]. Species that are highly sensitive to urbanization-related changes and are unable to survive in urban areas are called 'urban avoiders' [27]. On the opposite, some species benefit from these anthropogenic impacts. Cities can be a refuge with abundant human food and a release from biotic pressures such as predation and competition [59, 112]. Certain species have successfully adapted and flourished in urban environments. These species attain high population densities in cities [81, 101]. When they specialize to the point of living at the expense of humans, they are named urban dwellers [27]. Species with the ability to adapt to a wide range of environments and resources [87] are named urban adapters [27]. These species tolerate urban conditions, in particular green areas, but also survive in rural environments.

Understanding why and how some species adapt to urbanization is pivotal to addressing issues related to biodiversity crises [103]. Urbanization can trigger changes within species, driven by processes such as selection, epigenetic inheritance, and phenotypic plasticity [52, 78]. Recently, Alberdi et al. [1] advocated for a critical role of the microbial community in promoting host adaptation to rapid environmental changes, especially through its impact on host phenotypic plasticity. On one hand, the bacterial composition of the gut microbiota (noted “GM” hereafter) impacts animal biology and evolution by providing or influencing essential services that contribute to its health [84] including nutrition and metabolism [80], immunity [6] or behavior [24]. On the other hand, the GM is shaped by host characteristics (e.g. genomics, age or sex, [8, 83]), environmental features (e.g. climatic factors, resources, [29, 113]) and their interactions [105]. In addition, the GM can respond rapidly to environmental changes due to microbial flexibility (*i.e.* the adaptability or responsiveness of the microbial community through changes in taxonomic diversity and/or composition, [104]) but also to bacterial short-generation time and high mutation rate.

Urbanization may lead to various alterations in the bacterial gut microbiome by influencing the ecological processes that govern the assembly of bacterial communities, among which ecological drift, dispersion and selection [12]. Neutral changes are observed when the initial GM community is constant from a taxonomic or functional point of view [68]. Such changes could increase hosts’ phenotypic plasticity, which may be a prerequisite for adaptation [1].

Adaptive changes may occur as a consequence of variations in bacterial taxa driven by their relative ecological fitness, subsequently leading to changes in the composition and diversity of GM. These changes can allow hosts to survive in new ecological niches, notably through the ability to digest new food sources, to improve metabolic capacities or to increase tolerance to deleterious environmental conditions [64]. Over several generations, selection may favor hosts with advantageous GM, especially in disrupted environments [1, 68]. In the long term, there may be congruence between the evolutionary history of various host species and the community structure of their associated bacterial microbiomes, which is named phylosymbiosis [11, 49].

Lastly, maladaptive changes may also occur. They are associated with the disruption of GM homeostasis. This state is named dysbiosis [39] and has negative impacts on host fitness and health through alterations in the qualitative and quantitative composition of the GM or modifications in their metabolic functions [34]. Biomarkers of dysbiosis include a decrease in GM alpha diversity and higher heterogeneity of microbial composition between hosts [74], what has been observed in several studies analyzing the impacts of anthropogenic disturbances on GM in wildlife [25, 54, 114]. The Anna Karenina principle (AKP), which is related to gut microbiome dispersion and stochastic assembly (*i.e.* random changes in the establishment or extinction of bacterial taxa), proposes a framework based on these characteristics to detect dysbiosis*.* All healthy, balanced microbiota are similar, while disrupted microbiota are all different [117]. Under the AKP hypothesis, the level of microbiome dispersion and stochastic assembly may therefore reflect dysbiosis. This can lead to immune responses and metabolism dysregulation [70], consequently impacting negatively the hosts’ health [10].

Predicting the impact of environmental disturbances, especially urbanization, on wildlife and microbiota has been the topic of several studies this last decade. However, many of these studies have focused on a single host species (e.g. [25, 94, 95, 98, 107]). The few studies that investigated GM variations within host communities led to incongruent patterns with either a stronger impact of host phylogeny over habitats on GM assembly (e.g. [48]) or the opposite [97]. Therefore, gathering more data on the relationships between urbanization, host community assembly and their GM remains critical in the domain of urban ecology [103].

Small mammals constitute a relevant model to test hypotheses regarding these relationships. These animals represent a large diversity of mammals, have colonized a wide array of habitats and exploit diverse foraging niches [89]. Several rodents and insectivores occur in urban environments as urban adapters or urban dwellers. Besides, a recent meta-analysis performed by Santini et al. [82] has revealed that high diet diversity was a main factor predicting rodent adaptation to urbanization. This suggests that the GM could be at the core of this adaptation to urban environment.

**Fig. 1:**
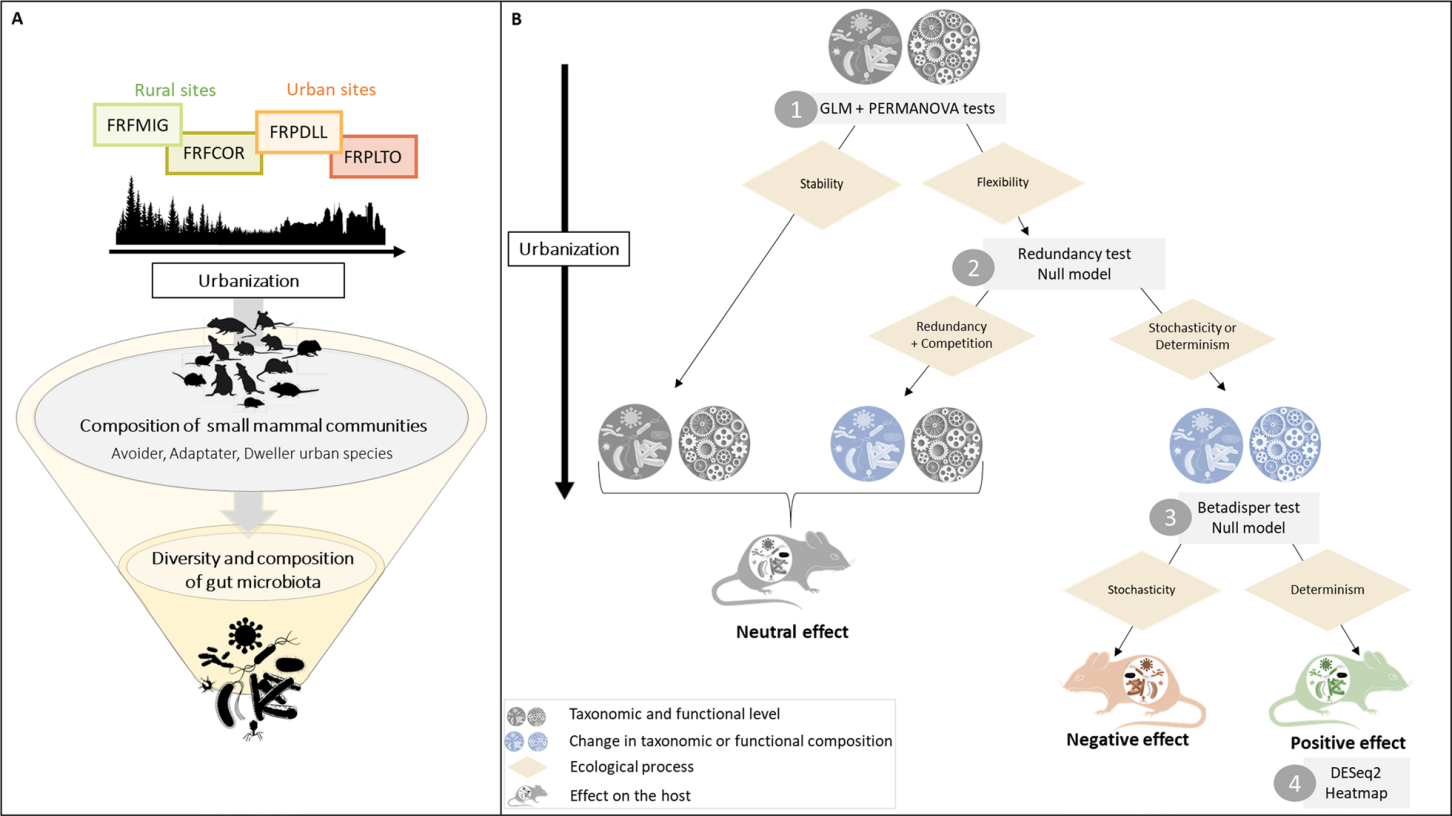
**A** We are testing the impact of urbanization on the composition of small mammal communities. Additionally, we are investigating whether the diversity and composition of the bacterial gut microbiota are influenced by the sites sampled along the urbanization gradient or by the small mammal species in presence. **B** Decision tree for interpreting changes in bacterial composition to highlight the processes that may influence the bacterial gut microbiota and its response to urbanization. Taxonomic and functional compositions are represented by icons, with blue indicating a change and grey an absence of change. Yellow diamonds represent ecological processes, and grey rectangles indicate the statistical tests performed to infer underlying ecological processes. Step 1. The GLM & PERMANOVA tests assesses whether there is a change in GM diversity and composition between sites that could be associated with urbanization. If there is no taxonomic change, it suggests that the bacterial gut microbiota remains stable despite urbanization, whereas if there are changes, the GM is considered to be flexible. When taxonomic changes occur, but functional changes are not necessarily present or are weak, redundancy processes may underlie the assembly of microbial communities. Step 2. Redundancy analysis is applied to identify situations where bacterial taxa express the same function (considered redundant) or where each taxon expresses its own function (not redundant). This test is followed by a null model test (NTI) to determine the mechanisms that may underlie the redundancy. These mechanisms can be selective processes through overdispersion (indicating competition between bacterial taxa) or underdispersion (indicating cooperation between bacterial taxa), resulting from abiotic or biotic effects among bacterial taxa. Additionally, it is worth noting that phylogenetic dispersion between bacterial taxa can also be influenced by neutral effects. Otherwise, when taxonomic and functional changes occur, stochastic or deterministic processes may underly GM assembly. Step 3. The betadisper test examines differences in intragroup variances. High variance may result from strong selective pressures, such as dietary variance, or stochastic effects indicating processes favoring dysbiosis (see Anna Karenina’s principle, [117]). Conversely, healthy species tend to express similar essential functions, resulting in lower variance. The null model helps to determine whether GM assemblage is primarily driven by stochasticity or determinism. Step 4. We use DESeq2 test to identify the functions that may be subject to selection. A heatmap enables to illustrate these functional changes between different host species and sites, reflecting the different levels of urbanization. The colored rodent icon indicates the likely impact of GM changes on its health. Grey represents a neutral effect, red a detrimental effect and green a beneficial effect

Here, we have investigated the interlinkages between urbanization, small mammal communities and their bacterial gut microbiome in forested areas. We analyzed the presence and abundance of rodents and insectivore’s species and the composition of their GM in different sites within rural and urban forests. We first checked that these sites mostly differed by their levels of urbanization, and we verified that small mammal species could be categorized as urban avoiders, adapters, or dwellers based on their distribution. Secondly, we investigated the variation in GM considering different factors, including host species, sampling sites, and individual features. We performed community ecology analyses to examine the differences in the composition and diversity of GM (1) between host species categories of urbanization response with regard to urban adaptation and (2) within host adapter species between sites. Next, we inferred the ecological processes (neutral and selective ones) that could underly the variations observed in GM composition and assembly, as well as the potential impacts of GM changes for the hosts (e.g. neutral, adaptative or maladaptive), following the decision tree detailed in Fig. 1. Overall, this study enabled to emphasize potential links between GM and small mammals’ responses to urbanization.

## Material and methods

### Data collection

#### Sampling and characterization of small mammals

We trapped small terrestrial mammals in four sites in Eastern France, in autumn 2020 (Additional file 1: Fig. S1). Two of them were located within the extensive rural forests of the French departments Ain (FRFCOR: Cormaranche en Bugey, 200.000 ha) and Jura (FRFMIG: Mignovillard; 250.000 ha). These sites were separated by approximately 140 km. The two other sites were forested areas within urban parks located in the Lyon metropolis (1.5 millions inhabitants). One park was situated in the heart of the city (FRPLTO: Lyon, Parc de la Tête d’Or; 105 ha) while the other one was a peri-urban park surrounded by residences but still connected to the forest (FRPDLL: Marcy l'étoile, Domaine Lacroix Laval; 115 ha). The distance between these two parks is approximately 20 km, and they are both around 150 km away from the two forested sites. More detailed information concerning the sampling design, capture results, and individual information were detailed in Pradel et al. [75]. Sexual maturity was determined a posteriori based on morphological and sexual characters. Animal trapping and sample collection were conducted according the EU Directive 2010/63/EU for animal experiments, as described in Pradel et al. [75].

The trapping success, often utilized as an indicator of relative rodent abundance when employing lethal traps, was estimated by analyzing the capture results obtained from the initial three nights of trapping [75]. It was calculated following Aplin et al. [4] as ln(1-number of rodent trapped /(number of traps × number of nights))x(-100) (Additional file 2: Table S1.1).

#### Environmental characterization of sampling sites

We characterized the level of site urbanization as well as other potentially distinguishing environmental features using Corine Land Cover ([18], 100 m of resolution) and Qgis software [43]. We extracted land cover area estimates for each site (Additional file 1: Fig. S1). Urbanization was quantified through the variables ‘Green urban areas’, ‘Industrial’ and ‘Continuous urban fabric’. In addition, we used Corine Land Cover of forests (10 m of resolution) to estimate forest fragmentation using the *Landscapemetric* package [37]. These calculations were made for each site by considering a 3 km buffer around the barycenter of the traps. Lastly, we extracted bioclimatic indices from the CHELSA database (1 km of resolution, https://chelsa-climate.org/,[43]) for each site using the coordinates of the traps’ barycenter (Additional file 2: Table S1.2).

#### Gut microbiota sequencing

For each individual, we extracted DNA from a 5 mm piece of colon tissue (lumen was removed) with the DNeasy 96 Blood and Tissue kit (Qiagen). We followed the manufacturer's instructions, with the exception of the addition of a bead-beating for 5 min with 500 mg of 0.5 mm zirconia beads in a TissueLyser (Qiagen) after the proteinase K digestion, as recommended in [17]. We amplified the V4 region of the 16S rRNA gene by PCR with the primers 16S-V4F [GTGCCAGCMGCCGCGGTAA] and 16S-V4R [GGACTACHVGGGTWTCTAATCC]) following the PCR conditions and program described in Galan et al. [28] and Kozich et al.  [51] Various controls were included to facilitate bioinformatics sorting of the sequences, including replication of libraries for all samples as well as negative controls for extraction, PCR and indexing, and the ZymoBIOMICS Microbial Community Standard (Zymo). We performed a run of 2 × 251 bp MiSeq paired-end sequencing. Information about the sequencing of samples and the raw sequence reads are detailed in the Zenodo repository (https://zenodo.org/record/8143272). In particular, the number of reads per sample after denoising is detailed in the file “Sample_Informations”.

#### Sequence processing

Amplicon sequence variants (ASVs) were generated using dada2 analysis pipeline (Qiime2_2021.11) [7, 13]. Both R1 and R2 reads were trimmed at 180 and 120 base-long respectively. This procedure improved the average quality of the reads (> Q30) and maximized the proportion of R1–R2 merging (approximately 80% of the total number of reads). Chimeric sequences were identified by the consensus method of the *removeBimeraDenovo* function. Taxonomic assignments were performed using blast +  implemented in the FROGS workflow [23] and the SILVA rRNA 138.1 database excluding the sequences with a pintail quality < 100 (http://www.arb175silva.de/projects/ssu-ref-nr/).

Further analyses were implemented in R v4.0.3 [77]. Scripts are available in Zenodo repository (https://zenodo.org/record/8143272). Sample metadata, abundance table, taxonomy table and tree are linked in the phyloseq object using the *Phyloseq* package [62].

We filtered false positives following the strategy described in Galan et al. [28]. In short, we discarded positive results with sequence counts below two ASV-specific thresholds, which checked respectively for (1) cross-contamination between samples or the presence of kitome during DNA extraction and PCR steps (T_CC_ for Threshold cross-contamination), and (2) incorrect assignment due to the generation of mixed clusters on the flowcell [47] during Illumina sequencing (T_FA_ for Threshold false-assignment). The T_CC_ corresponds to the maximum number of reads observed in a negative control for DNA extraction or negative control for PCR for each ASV. The T_FA_ corresponds to the putative maximum number of reads assigned by mistake to a wrong sample. We have chosen a *Mycoplasma capricolum* sample as an internal DNA control, with a maximum rate of read misassignment of 10^–4^ in all MiSeq sequencing runs conducted in our laboratory from 2020 to 2022. Finally, for each sample and each ASV, only the occurrences confirmed by the two technical replicates were kept in the dataset. At this stage, the reads of the technical replicates for each sample were summed.

Based on the taxonomy of ASVs, only the kingdom Bacteria was retained. The chloroplast phyla, the unaffiliated phyla and the *Mitochondria* family were removed. We filtered individuals based on the rarefaction procedure implemented in *Phyloseq*. Individuals for which the sequencing depth was insufficient to determine all the ASVs present (*i.e.* for which the plateau had not been reached) were deleted. The phylogenetic tree of ASVs was built after these filtration steps with FROGS using *FastTree* [76] and on the basis of a multiple alignment performed with *MAFFT* [45].

Next, we removed individuals corresponding to a low number of captures per site and species (threshold of five individuals/species/sites). We also excluded individuals found dead in the traps. Their GM composition differed from that of individuals captured alive, what could be explained by an advanced stage of degradation of the GM of these former individuals [57]. We also removed *Glis glis* individuals from the statistical analyses dedicated to GM, as the GM of this species exhibited an extremely low level of diversity with an overrepresentation of a particular ASV (Additional file 3).

ASVs number of reads were finally normalized to proportional abundance for each individual [61].

### Statistical analyses

#### Composition of small mammal communities

We primarily tested whether the sampling sites differed in their level of urbanization rather than being influenced by other potential confounding factors. We performed a multivariate analysis as recommended by Moll et al. [65]. After removing significant covariates detected by a Pearson correlation test, we performed a principal component analysis (PCA) with all environmental and bioclimatic features using *FactoMineR* package [55]. We verified that the first axis of the PCA explained a large portion of the abiotic differences between sites and effectively captured urbanization by contrasting sites with high artificial and urban environments vs sites with larger forest areas, which corresponds to our definition of urbanization. In this case, we designated PCA1 as the urbanization axis, aligning with the approach proposed by Du Toit and Cilliers [22].

Next, we analyzed whether small mammal communities’ composition differed between sites, and we verified that host species could be classified into three classical categories of urbanization response, namely avoiders, adapters and dwellers [27]. We utilized the Bray–Curtis index to generate a dissimilarity matrix of small mammals trapping success (abundance proxy) among sites. We tested whether the urbanization score influenced this dissimilarity matrix using the *capscale* function of the *vegan* package [72]. Significance was assessed using 10,000 permutations. We selected the best model using the *ordiR*^2^*step* function.

#### Gut microbiota taxonomic and functional diversity

Throughout this study, we described GM using information relative to bacterial taxa (*i.e.* ASVs) and functional metagenomic predictions obtained using *Picrust*2 software [20], NSTI < 2). For functional predictions, the metabolic pathway description and enzyme classes were obtained from the MetaCyc database [15].

#### Variations in the alpha diversity of the gut microbiota

We first estimated the GM alpha diversity, *i.e.* the diversity of within-host bacteria, using the taxonomic richness, the Shannon index and two metrics considering taxa phylogenetic diversity, estimated using the *picante* package [46]. These metrics are the Faith index which corresponds to phylogenetic diversity (PD) and the Nearest Taxon Index (NTI) [108], which reflects phylogenetic structuring near the ends of the tree. Finally, in order to address functional diversity, we calculated the richness index of predicted functions using *MiscMetabar* (e. g. [2, 79, 96]).

We next tested the relative influence of host species, sampling sites, and their interaction on the taxonomic and functional diversity indices described above using generalized linear models (GLMs). Urbanization per se could not be tested directly due to other potential abiotic factors distinguishing sites. Individual factors such as maturity and sex of animals were also investigated. We used the negative binomial distribution for species richness and the Gaussian distribution for the other indices. We highlighted the deviation and dispersion of the model residuals from normality with the *DHARMa* package [33] and we applied transformation when necessary. The selection of the best model was made by considering all possible model combinations using the *dredge* function of the *MuMIn* package and the Akaike information criterion corrected for small sample size AICc [41]. We then averaged the partitioned variance of each factor in the best model using *variancePartition* [38]. When site and/or species factors were significant, Tukey's post-hoc tests were applied to assess pairwise differences in modalities, using the *multcomp* package [40]. Because species were not evenly distributed among sites, the dataset was not balanced. To better assess the interaction effect, a factor combining species and site was defined (named species-site hereafter). A linear model was applied, followed by a Tukey post-hoc test comparing only possible biological interactions with a contrast matrix. Specifically, we compared the diversity (1) between host species within a given site and (2) within adapter species between sites to explore the potential effects of urbanization.

#### Ecological processes shaping the diversity of the gut microbiota

We used two approaches to infer the relative influence of ecological processes in shaping the GM taxonomic and functional diversity.

We examined the importance of functional redundancy by analyzing the correlations between bacterial functional and taxonomic richness for each species-site combination (Fig. 1B, step 2). A slope lower than 1 indicated functional redundancy, *i.e.* multiple bacterial taxa having the same function [53], while a slope greater than 1 indicated that bacterial taxa may have more than one function. We tested whether these slopes differed significantly between species-site combinations, using an analysis of covariance (ANCOVA) performed with a GLM. A post-hoc test was applied using *emmeans_test* with *rstatix* package [44] to compare pairs of slopes between species-site combinations. Here, we considered the taxonomic richness as a covariate of functional richness. The matrix contrast of biological pairs and Benjamini–Hochberg corrections for multiple tests were implemented to assess the significance of interactions.

Next, NTI values provided information about the relative influences of stochastic and deterministic processes on GM composition [109]. When GM is predominantly influenced by stochastic processes, its phylogenetic composition is expected to align closely with random community assembly expectations (− 2 < NTI < 2). In the opposite, selection mediated by environment (environmental filtering) should lead to phylogenetic clustering, *i.e.* the coexistence of taxa that are more closely related than expected by chance (NTI > 2). Selection mediated by bacterial species competitive interactions should lead to phylogenetic overdispersion, *i.e.* the coexistence of taxa that are more distantly related than expected by chance (NTI < − 2). We tested these hypotheses by comparing bacterial ASVs phylogeny to a null model, generated by randomizing the ASV labels between taxa (with the parameter: null.model = "taxa.labels"; Fig. 1B, step3).

#### Gut microbiota taxonomic and functional composition

##### Variations in the beta diversity of the gut microbiota

We measured GM beta diversity, which refers to bacteria diversity between hosts, to compare taxonomic and functional composition with regard to host species, sampling sites and individual features. Additionally, we examined (1) between host species categories of urbanization response and (2) within host adapter species between sites.

Dissimilarities in taxon composition (ASVs) were calculated based on the normalized ASVs abundance table and the weighted unifrac index. This index considers both the abundance of ASVs and their phylogenetic relationships. Results gathered from other indices (Jaccard, Bray–Curtis and Unifrac are presented in Additional files only). We analyzed changes in GM composition between sites using the *vegan* package [72]. We tested the influence of small mammal species, sites and individual factors (sex and maturity) on GM composition using a permutational analysis of variance (PERMANOVA) implemented with the *capscale* function. The best model was selected using permutation tests in constrained ordination, and the *ordiR*^2^*step* function maximizing the *R*^2^ value. Besides, we performed a distance-based redundancy analyses (db-RDA) from the previously obtained constrained ordination. This enabled to highlight, in addition to the “*pairwise adonis*” post-hoc test, the variation between each pair within sites and species.

##### Ecological processes shaping the composition of gut microbiota

We applied several approaches to infer the relative influence of ecological processes in shaping the composition of the GM (Fig. 1B).

First, PERMANOVA analyses were performed to provide information on GM flexibility (Fig. 1B, step 1). We considered both variations in GM composition between sites for adaptive species, to explore the potential effects of urbanization, and variations in GM composition between sympatric urban adapter and dweller species.

Second, we tested whether the variations in GM composition resulted from adaptive or non-adaptive changes. We estimated intra-host species and intra-site dispersion using the *PERMDISP2* test implemented with the *betadisper* function. High dispersion indicated that the GM composition was likely to be driven by stochastic processes while low dispersion could reflect selective processes favoring similar microbial functions (Fig. 1B, step 3).

In addition, the null model approach was applied to quantify the contribution of ecological processes (stochasticity vs selection) on GM composition assembly and turnover [92]. We followed the procedure described by Barnett et al. [5] to generate the β-nearest taxon index (βNTI). Briefly, we used the *comdistnt* function from the *picante* package to determine the β-mean-nearest taxon distance (βMNTD). We generated null values of βMNTD by randomly reshuffling 1,000 times the extremities of the phylogenetic tree. Finally, βNTI was calculated according to the formula: βNTI = (βMNTDobserved-mean (βMNTD null))/ standard deviation (βMNTDnull). Selection was a major process when βNTI was lower than −2 or higher than 2 [92]. In this case, the phylogenetic turnover of the GM was lower or higher than expected by chance respectively. In contrast, when βNTI values ranged between −2 and 2, we concluded that stochastic processes were the main drivers of the GM composition.

After clustering individuals by species-site combination, we counted the number of observations of each ecological process, according to the βNTI values, within each combination. We performed a chi-2 test to detect observations that were significantly different from null expectations.

Lastly, we determined whether individuals from a given species-site combination had more similar functions than individuals from different species-site combinations, which could be the result of selective processes (Fig. 1B, step 4). This analysis was performed using the *DESEq2* package with count data and a negative binomial family (the Wald Test parameter) [58]. We added + 1 to the abundance dataset to avoid a zero-inflation bias.

## Results

### Urbanization influences small mammal communities

Based on a sampling effort of 2163 traps-nights, we recorded a total of 228 small mammals*, i.e.* a global trapping success rate of 10.5%. These individuals corresponded to 14 species. Nine species belonged to the order *Rodentia* and to four families: *Gliridae* (*Glis glis*), *Sciuridae* (*Sciurus vulgaris*), *Muridae* (*Rattus norvegicus, Mus musculus*, *Apodemus sylvaticus* and *Apodemus flavicollis*) and *Cricetidae* (*Myodes glareolus, syn. Clethrionomys glareolus*, *Microtus arvalis* and *Microtus agrestis*). Five species belonged to the order *Soricomopha* and *Soricidae* family (*Sorex araneus*, *Sorex coronatus*, *Neomys fodiens*, *Crocidura russula* and *Crocidura leucodon*) (Additional file 4: Fig. S2.1).

#### Sampling sites differ by their level of urbanization

**Fig. 2:**
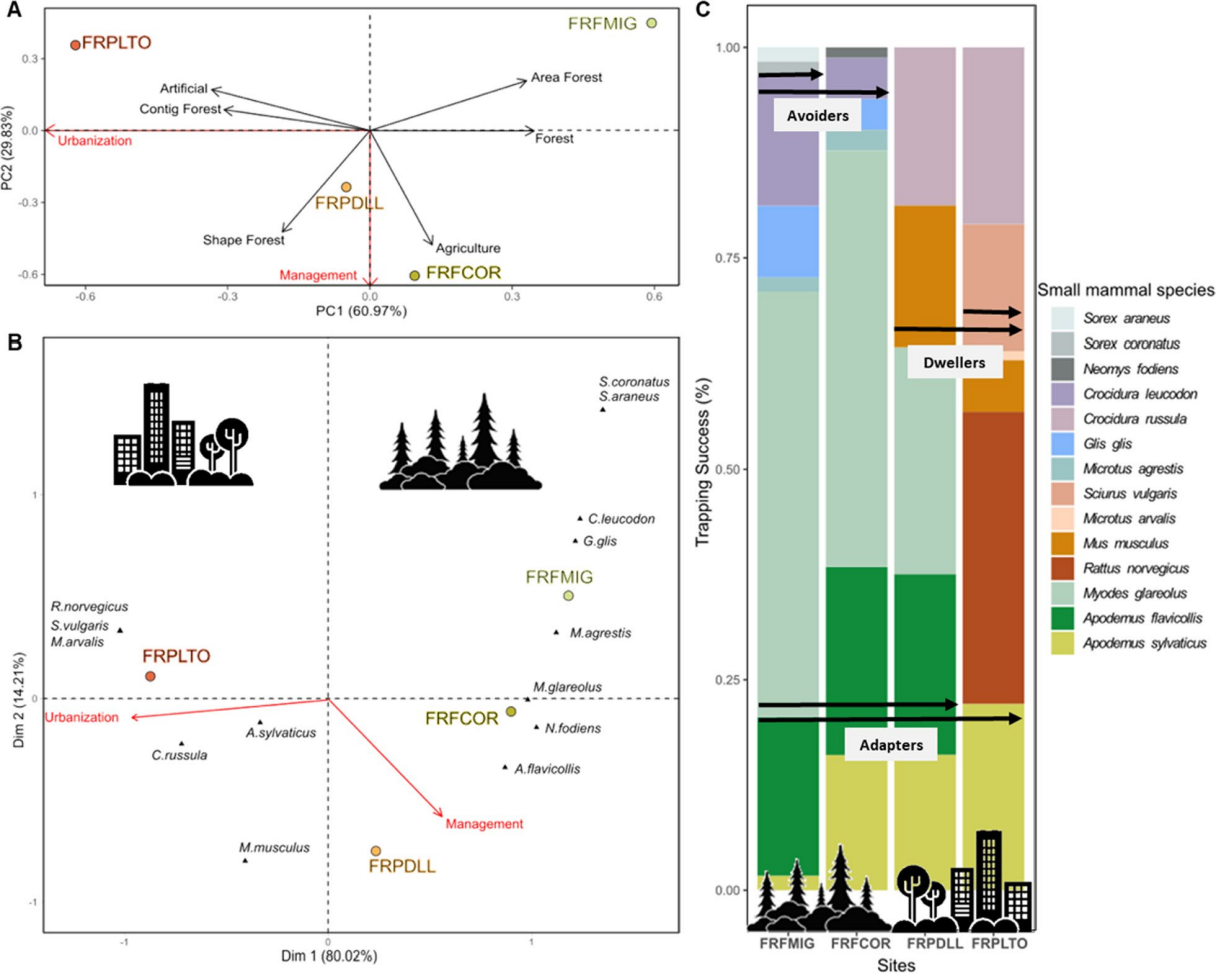
Analyses of small mammal community variations. **A** Principal component analysis (PCA) of the environmental features characterizing sampling sites (black arrows. The red arrows indicate that PCA1 axis describes different levels of urbanization while PCA2 axis describes differences in land use management and resource exploitation. **B** Canonical correspondence analysis (CCA) of the small mammal trapping success per site. Sites are represented by dots and color code. Species are represented by a black triangle. The two first PCA axes were included as explanatory variables in the canonical analysis and are represented by red arrows. **C** Bar graph showing the relative trapping success of species (represented by colors) per site, ordered from the less (FRFMIG) to the more (FRPLTO) urbanized ones. The icons highlight the variations between urban and rural locations, with arrows denoting the species classification on the urbanization spectrum. Species that are not found in urban zones are classified as urban avoiders, those only present in urban areas are urban dwellers, and those found in both areas are urban adapters

The first axis of the PCA based on site characteristics explained 60.97% of the total variation and contrasted sites with high artificial and urban environments vs sites with larger forest areas (Fig. 2A), which corresponds to our definition of urbanization (more settlements and artificialization). Sampling sites were organized along this axis, reflecting their different levels of urbanization, and corroborating the opposition between urban sites (FRPLTO and in a lesser extent FRPDLL) and rural sites (FRFCOR and FRFMIG) despite potential other confounding factors.

#### Small mammal species exhibit distinct categories of response to urbanization

The assembly of small mammal communities differed among sampling sites (Fig. 2A; Additional file 4: Fig. S2.2). Although the geography, climate and land use explained part of this variation (Additional file 1: Fig. S1; Additional file 2: Table S1.2), urbanization was the main factor driving the differences observed between sites. Rural sites homed seven (FRFCOR) or eight (FRFMIG) small mammal species and they had six species in common. Urban sites homed fewer small mammal species (respectively five in FRPDLL and six in FRFLTO) and they shared three species. FRFDLL also had three species in common with the rural sites, while FRFPLTO had only one species in common with these sites. The level of sharing of small mammal species among sites is detailed in Additional file 4: Fig. S2.2.

The CCA based on small mammal relative abundance revealed that sites differed in the composition of small mammal communities and this variation was found to be associated with urbanization (Fig. 2B). The first axis of the CCA explained 80% of the total variance and it represented the urbanization score. PERMANOVA tests showed that small mammal community composition was significantly influenced by PCA1 coordinates, *i.e.* the score of urbanization (F = 5.36, p = 0.04) with 57% of the variance explained by this factor (Additional file 2: Table S1.4).

Small mammal species corresponded to the avoider, adapter and dweller categories defined in the literature to describe wildlife responses to urbanization [27]. Some species, *G. glis*, *M. agrestis*, *S. araneus*, *S. coronatus*, *N. fodiens* and *C. leucodon,* were urban avoiders. Their relative abundance was always lower than the urban dweller and adapter species. Other species were present in both rural and urban sites. These urban adapters are *M. glareolus*, *A. flavicollis* and *A. sylvaticus* (Fig. 2C). They varied in abundance between sites: the relative abundance of *A. sylvaticus* increased along the urbanization gradient while the relative abundance of *M. glareolus* and *A. flavicollis* decreased with urbanization. They were even absent from the most urbanized site FRPLTO. In contrast, urban dwellers, namely *R. norvegicus*, *M. musculus*, *S. vulgaris* and *C. russula,* were relatively abundant compared to urban adapters.

Most avoider urban species exhibited very low abundance, even in rural forests (*Neomys fodiens, Sorex araneus, S. coronatus, Crocidura leucodon*). All these species are categorized into the ‘low concern’ category in the IUCN red list of threatened species in France, although *N. fodiens* is classified as ‘vulnerable’ in some parts of Eastern France (Alsace). Given their rarity, these species were excluded from subsequent analyses of GM diversity and composition, with the exception of *C. leucodon.*

In urban parks, the number of *Microtus arvalis, M. agrestis* and *Sciurus vulgaris*, per site was lower than 5, mostly due to field constraints, so we removed these species from further microbiome analyses.

### Urbanization influences alpha diversity of small mammal gut microbiota

After the filtration steps, we obtained a total of 5478 ASVs from 222 small mammals (Additional file 5: Fig. S3), resulting in 1969 enzyme commissions (ECs) and 383 metabolic pathways. For all analyses, we found similar results between EC and metabolic pathway diversity, so we only presented the results relative to pathways below.

#### Small mammal species identity influences gut microbiota alpha diversity (Fig. 1B, step1)

**Fig. 3:**
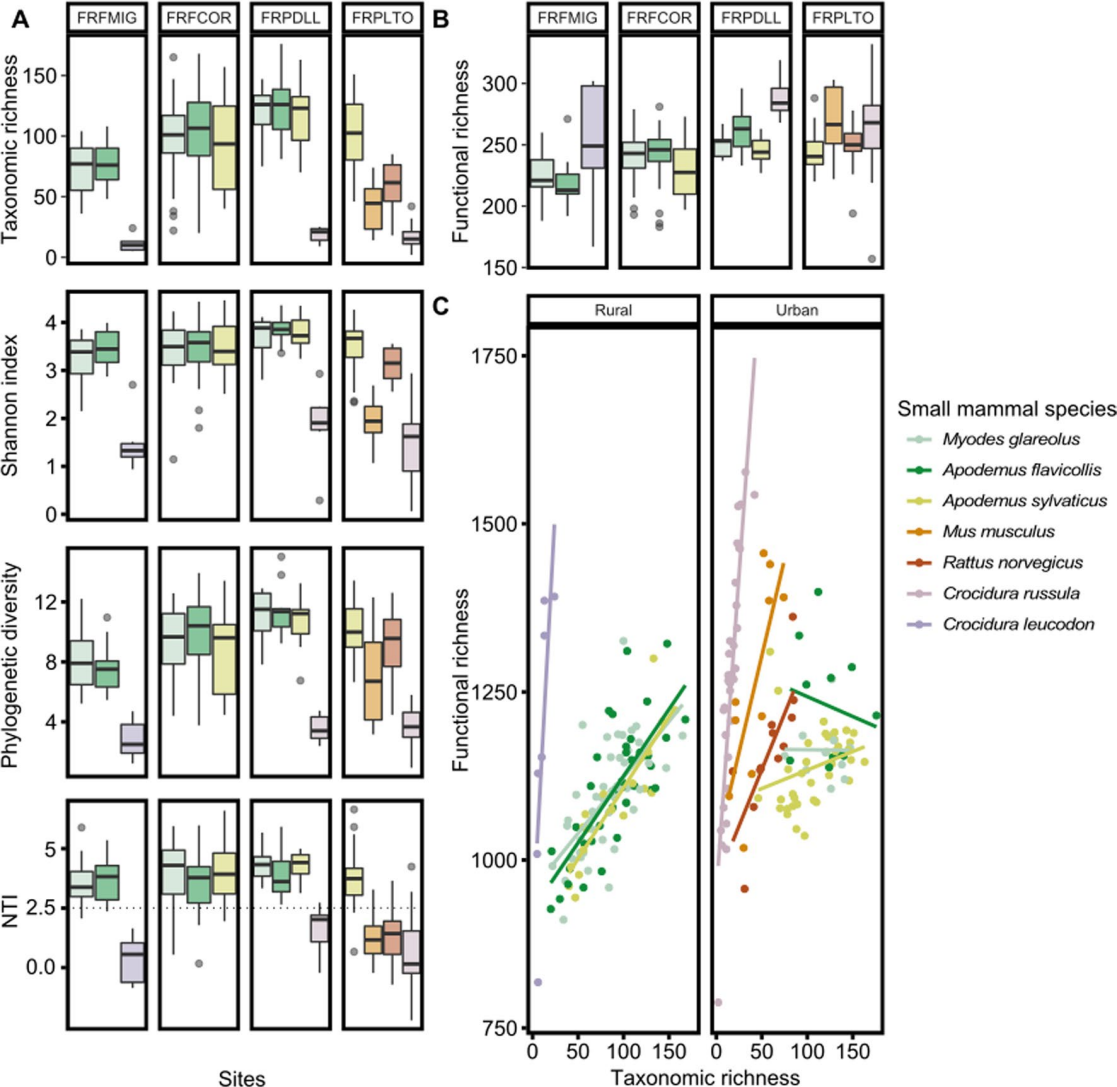
Boxplot showing variation in the alpha diversity of the gut microbiota between small mammal species in rural sites (sites FRFMIG and FRFCOR) and urban sites (sites FRPDLL and FRFLTO). The color codes correspond to small mammal species and they remain the same for all subfigures. Alpha diversity was measured **A** at the taxon level with the taxonomic richness (number of ASVs), Shannon index, phylogenetic diversity (PD) and Nearest Taxon Index (NTI) and **B** at the functional level with the functional richness corresponding to the number of metabolic pathways. **C** Plot of the correlation between the taxonomic and functional richness for each small mammal species in rural (FRFMIG, FRFCOR) and urban (FRPDLL, FRPLTO) sites

The analyses were conducted on the seven small mammal species with a sufficiently large effective (*Myodes glareolus, Apodemus flavicollis, A. sylvaticus, Mus musculus, Rattus norvegicus, Crocidura russula and Crocidura leucodon*, see details in Additional file 5: Fig. S3). Small mammal species was the main factor explaining variations in GM alpha diversity, whatever the indices analyzed (GLM, taxonomic richness: *F* = 80.42, *p* =  < 2.2e−16; Shannon index: *F* = 56.75, *p* = 2.2e−16; Faith’s PD: *F* = 37.58, *p* = 2.2e−16, Fig. 3A; functional diversity: *F* = 7.29, *p* = 4.4e−07; Fig. 3B; Table 1).

**Table 1:**
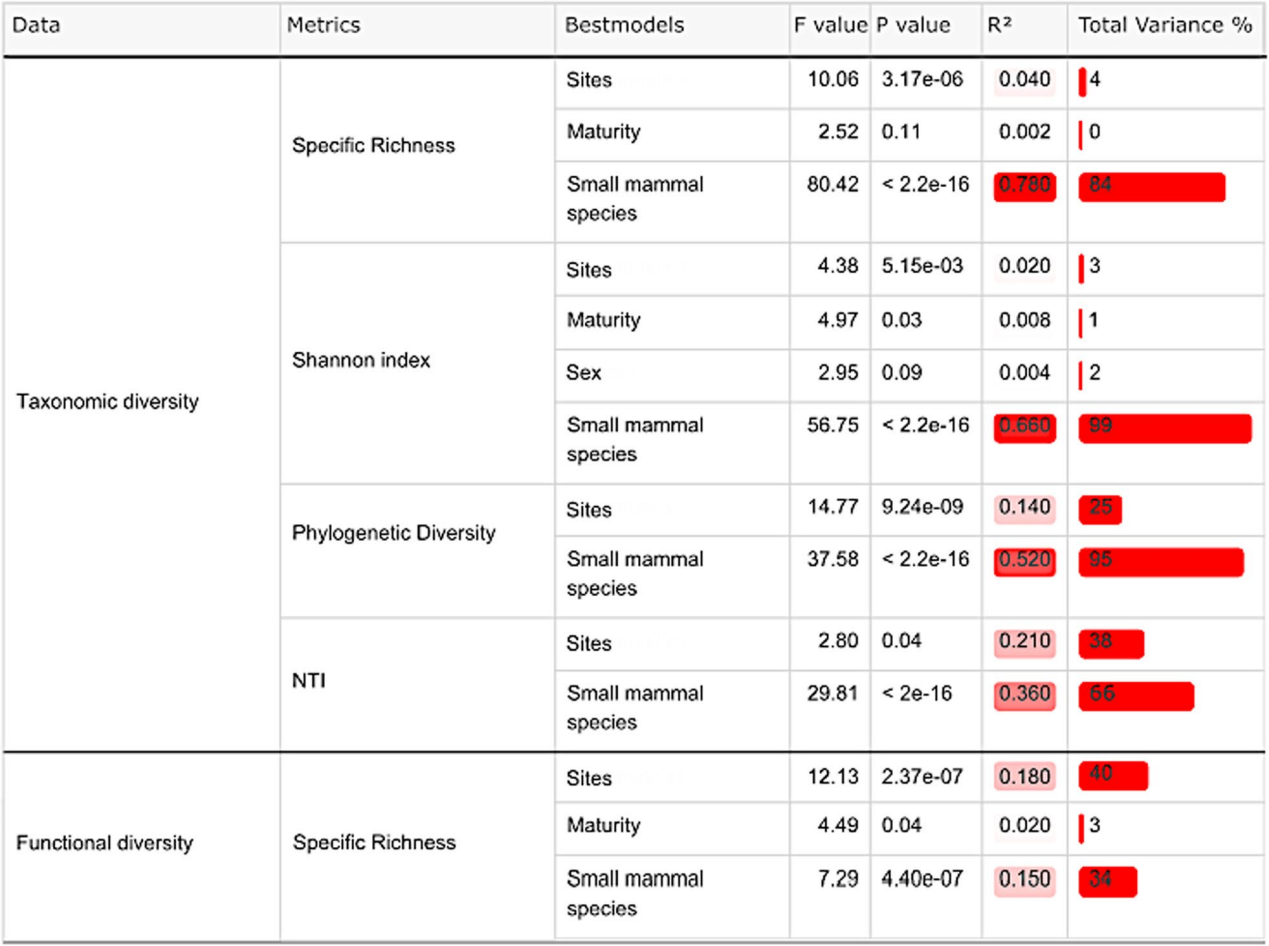
Results of the best models for the different GLM tests performed for each taxonomic and functional diversity index

For each variable in the best model, the *F* value, the *P* value, R^2^ and the percentage of the variance explained are indicated (some factors explain the same proportion of the variance). The red gradient indicates the importance of the R^2^ and of the variance explained in the models

Among *Muridae*, the GM of the three urban adapter species, *A. sylvaticus*, *A. flavicollis*, and *M. glareolus*, exhibited similar levels of alpha diversity, whatever the indices considered (Fig. 3; Additional file 6: Table S2.1).

The GM taxonomic diversity was significantly lower for urban dweller species than for urban adapter species (Fig. 3A; Additional file 6: Table S2.1). *C. russula*, *M. musculus* and *R.* *norvegicus* had lower taxonomic diversity than *Apodemus* sp. and *M. glareolus* in FRPDLL and/or FRPLTO. This pattern was less pronounced for *R. norvegicus* in FRPLTO when considering Shannon and PD indices compared to the taxonomic richness (Fig. 3A, Additional file 6: Table S2.2).

Conversely, the urban dweller species showed a greater GM functional diversity than the urban adapter species (e.g. *M. musculus*, *R. norvegicus* and *C. russula* compared to *A. sylvaticus* in FRPLTO, or *C. russula* compared to *Apodemus* sp. and *M. glareolus* in FRPDLL; Fig. 3B; Additional file 6: Table S2.2).

#### Gut microbiota alpha diversity differs between sites (Fig. 1B, step1)

Overall, the GM diversity changed between sites for all diversity indices considered except NTI, with a maximum level reached in FRPDLL (Fig. 3A and B, Additional file 6: Table S2.1). The GM diversity of adapter species was lower in the rural sites FRFMIG and FRFCOR than in the urban ones FRPLTO and FRPDLL (Additional file 6: Table S2.1). This potential impact of urbanization was dampened when considering the Shannon index (Additional file 6: Table S2.1).

The interaction between species and site significantly explained variations of alpha diversity in all the models tested (GLM; Taxonomic richness: *F* = 43.86, *p* < 2.2e−16; Shannon: *F* = 33.67, *p* = 2.2e−16; PD: *F* = 21.39, *p* < 2.2e−16; Functional richness (Pathway): *F* = 9.27, *p* = 5.58e−15; Additional file 6: Table S2.2). The GM alpha diversity of *M. glareolus* and *A. flavicollis,* but not *A.* *sylvaticus,* varied between sites, showing maximum values for the peri-urban park FRPDLL, whatever the indices considered (Additional file 6: Table S2.2).

#### Gut microbiota functional redundancy and phylogenetic clustering vary between sites (Fig. 1B, step2)

Functional redundancy was analyzed through the covariance between functional and taxonomic richness indices. We found that it was significantly influenced by small mammal species (ANCOVA, *F* = 19.71, *p* = 2.2e−16), site (ANCOVA, *F* = 6.71, *p* = 2 × 10^–4^) and sex (ANCOVA, *F* = 5.26, *p* = 0.02) (Fig. 3C). Overall, the covariance was higher for urban dweller species than for urban adapter species, suggesting a lower level of functional redundancy for urban dwellers (Fig. 3C; Additional file 6: Table S2.3). This pattern was also significant at the scale of a particular site. For example, at FRPLTO, the covariance between functional and taxonomic richness indices for all urban dweller species was higher than 1 (*R. norvegicus, a* = 3.30; *M. musculus, a* = 5.70; *C. russula, a* = 17.00) whereas it was lower than 1 for *A. sylvaticus* indicating functional redundancy for this urban adapter species (*A. sylvaticus, a* = 0.37).

The covariance between functional and taxonomic richness indices changed between sites for urban adapter species, with high levels of functional redundancy in urban parks but not in rural forests (Additional file 6: Table S2.3; Fig. 3C; *M. glareolus*: FRFMIG, *a* = 2.10, FRFCOR, *a* = 1.90 and FRPDLL, *a* = −0.02; *A. flavicollis*: FRFMIG, *a* = 2.10, FRFCOR, *a* = 1.90; FRPDLL, *a* = −0.59; *A. sylvaticus*: FRFCOR, *a* = 2.10, FRPDLL, *a* = 0.93 and FRPLTO, *a* = 0.37).

NTI showed significant variations mainly among species categories of urbanization response (*F* = 29.8, *p* = 2.2e−16). We detected high NTI values for urban adapter species (NTI < −2), indicating a significant phylogenetic clustering (Fig. 3A. Additional file 6: Table S2.1). No significant pattern was detected for the urban dweller species (NTI values ranged between − 2 and 2, close of 0). No changes were observed between sites for the adapter species (Additional file 6: Table S2.1).

### Gut microbiota composition changes with urbanization

#### Gut microbiota composition varies between sites (Fig. 1B, Step1)

The taxonomic composition of the GM, as summarized with beta diversity indices, was significantly influenced by small mammal species (PERMANOVA, *R*^2^ = 0.32, *F* = 12.57, *p* = 0.001) and sites (PERMANOVA, *R*^2^ = 0.08, *F* = 9.06, *p* = 0.001) (Additional file 7: Table S3.1). We observed the same results for the GM functional composition (PERMANOVA, species: *R*^2^ = 0.25, *F* = 8.50, *p* = 0.001; sites: *R*^2^ = 0.08, *F* = 7.70, *p* = 0.001; Additional file 7: Table S3.1).

**Fig. 4:**
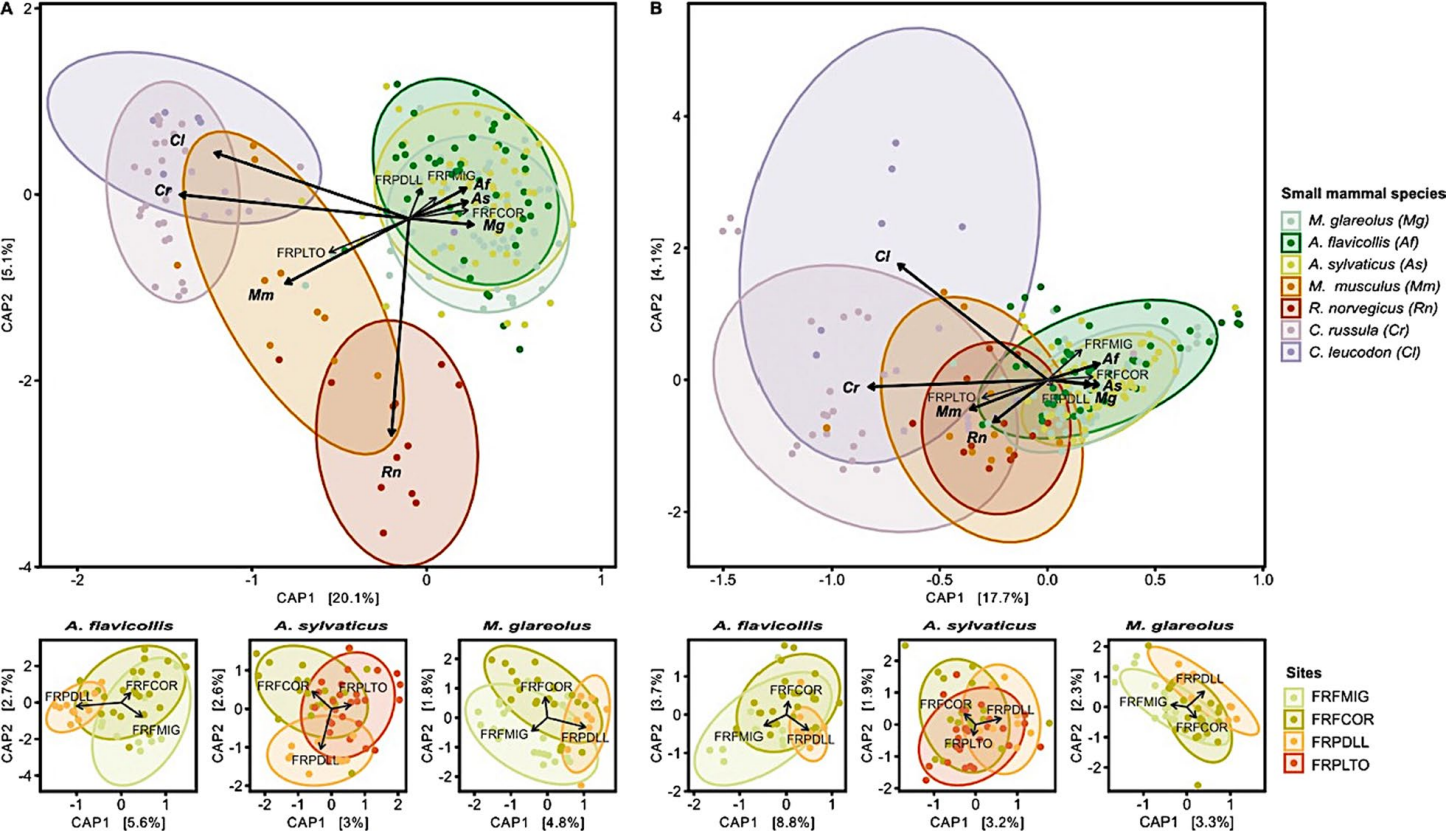
Distance-based redundancy analysis (db-RDA) of small mammal GM performed **A** on ASVs and using the weighted Unifrac dissimilarity matrix and **B** on functions and using the Bray–Curtis dissimilarity matrix. Only significant factors based on the *capscale* and *ordiR2step* analyses are indicated by arrows. The ellipses represent a 90% confidence interval around the centroids of the groups. The larger graph shows the significant factors modulating GM composition. Each point represents the GM of an individual and the color illustrates the small mammal species. The smaller graphs below represent the variation of the GM composition along the urbanization gradient for urban adapter species (*A. sylvaticus; A.flavicollis* and *M.* *glareolus*). Each point represents the GM of an individual and the colors represent sites and the urbanization gradient

The db-RDA (Fig. 4A, B) provided a more detailed picture of differences in GM composition between sites and species (See Additional file 8: Fig. S4 for results obtained with Jaccard, Bray–Curtis and Unifrac indices). We found that the clustering of individuals was driven by their species affiliation, then by their adaptation to urbanization rather than by their phylogeny (partial Mantel test, ASV: *R* = 0.61, *p* = 0.004; function *R* = 0.58, *p* = 0.004; Additional file 9: Fig. S5, Additional file 7: Table S3.2). Indeed, the first axes opposed urban dweller species to urban adapter species. The GM composition (ASVs and functions) of *A. sylvaticus* (family *Muridae*) was closer to the one of *M. glareolus* (family *Cricetidae*) than to the one of *R. norvegicus* or *M. musculus* (family *Muridae*) (Fig. 4). These differences were also observed when considering species living in sympatry, especially at FRPLTO (Additional file 7: Table S3.1, Additional file 8: Fig. S4).

Urban adapter species showed contrasted changes in GM composition between sites. No change in taxonomic nor functional composition was observed for *A. sylvaticus* (ASV: Fig. 4A, F = 1.60, *p* = 0.052; function: Fig. 4B, F = 1.46, *p* = 0.110). *A. flavicollis* GM composition slightly changed between sites at both the taxonomic (Fig. 4A, F = 2.03, *p* = 0.020) and functional (Fig. 4B, F = 3.22, *p* = 0.010) levels. *M. glareolus* GM changed between sites at the taxonomic level (Fig. 4A; *F* = 1.87, *p* = 0.020) but not at the functional level (Fig. 4B; *F* = 1.58, *p* = 0.090). Regardless of the species, the low variance accounting for differences in GM composition between sites indicates minimal spatial fluctuations in the GM composition (Fig. 4A, B).

The relative influence of ecological processes driving bacterial gut microbiota composition varied among sites (Fig. 1B, step3). We showed that the overall dispersion of the GM composition differed between sites (Betadisper test, ASV: *F* = 17.53, *p* = 3.16e−10; function *F* = 11.73, *p* = 3.78e−07, Additional file 10: Fig. S6), and reached higher values in the more urbanized park FRPLTO (Additional file 7: Table S3.3). Small mammal species differed significantly in terms of dispersion (Betadisper test, ASV: *F* = 6.67, *p* = 9.37e−08; function: *F* = 31.85, *p* = 2.2e−16). Urban adapter species exhibited lower levels of dispersion than some urban dwellers, mainly in urban parks (e.g. *C. russula* for both taxonomic and functional composition; *M. musculus* for the functional composition only, Additional file 7: Table S3.3).

**Fig. 5:**
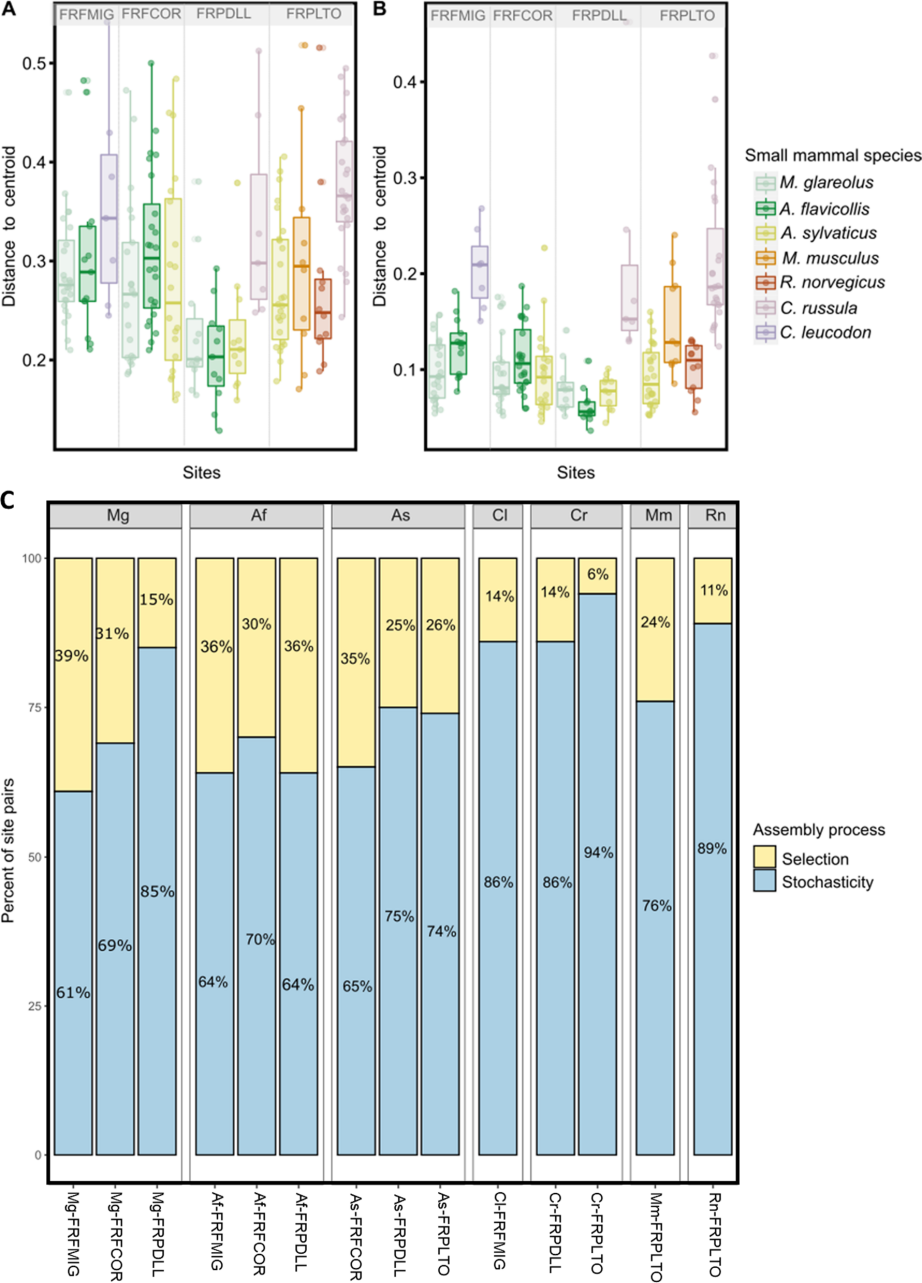
Boxplot of the distance to the centroid of each individual for each species-site combination at **A** taxonomic and **B** functional levels. Each point corresponds to an individual and the colors correspond to small mammal species. The combinations are ordered according to the urbanization gradient. **C** Processes responsible for bacterial assembly and turnover in each combination of small mammal species and site. Percentage of pairs of individuals within each species-site combination for which selective processes (yellow) or stochastic processes (blue) are emphasized

When we considered species-site combinations, we showed that the dispersion in GM composition was higher in rural sites than in the urban park FRDLL for two urban adapter species *M. glareolus* and *A. flavicollis* (Betadisper test, ASV: *F* = 5.06, *p* = 1.77e−06, Fig. 5A; function: *F* = 16.30, *p* = 2.2e−16, Fig. 5B; Additional file 7: Table S3.3).

We next assessed the relative influence of community assembly processes using the βNTI index. Phylogenetic turnover was mainly shaped by stochasticity (> 60%) (Fig. 5C). The allocation of assembly processes within species-site combinations was significantly different from what was expected (Chi-2 test, *c*^2^ = 120.5, *p* = 2.2e−16). The “small mammal species” and “sampling sites” factors had a significant effect on GM assembly. The GM assembly of urban adapter species was relatively more influenced by selection in rural forests (FRFMIG: *M. glareolus* 39%, *A. flavicollis* 36%; FRFCOR: *M. glareolus* 31%, *A. flavicollis* 30% and *A. sylvaticus* 35%), as well as in the urban park FRPDLL for *A. flavicollis* (36%) (Fig. 5C, Additional file 11: S7). We detected a strong influence of stochasticity in dweller species (*C. russula* in FRPDLL and FRPLTO, 86% and 94%, respectively; *R. norvegicus* in FRPLTO, 89%), avoider species (*C. leucodon* in FRFMIG, 86%) and for *M. glareolus* in the urban park FRPDLL (85%) (Fig. 5C, Additional file 11: S7).

**Fig. 6:**
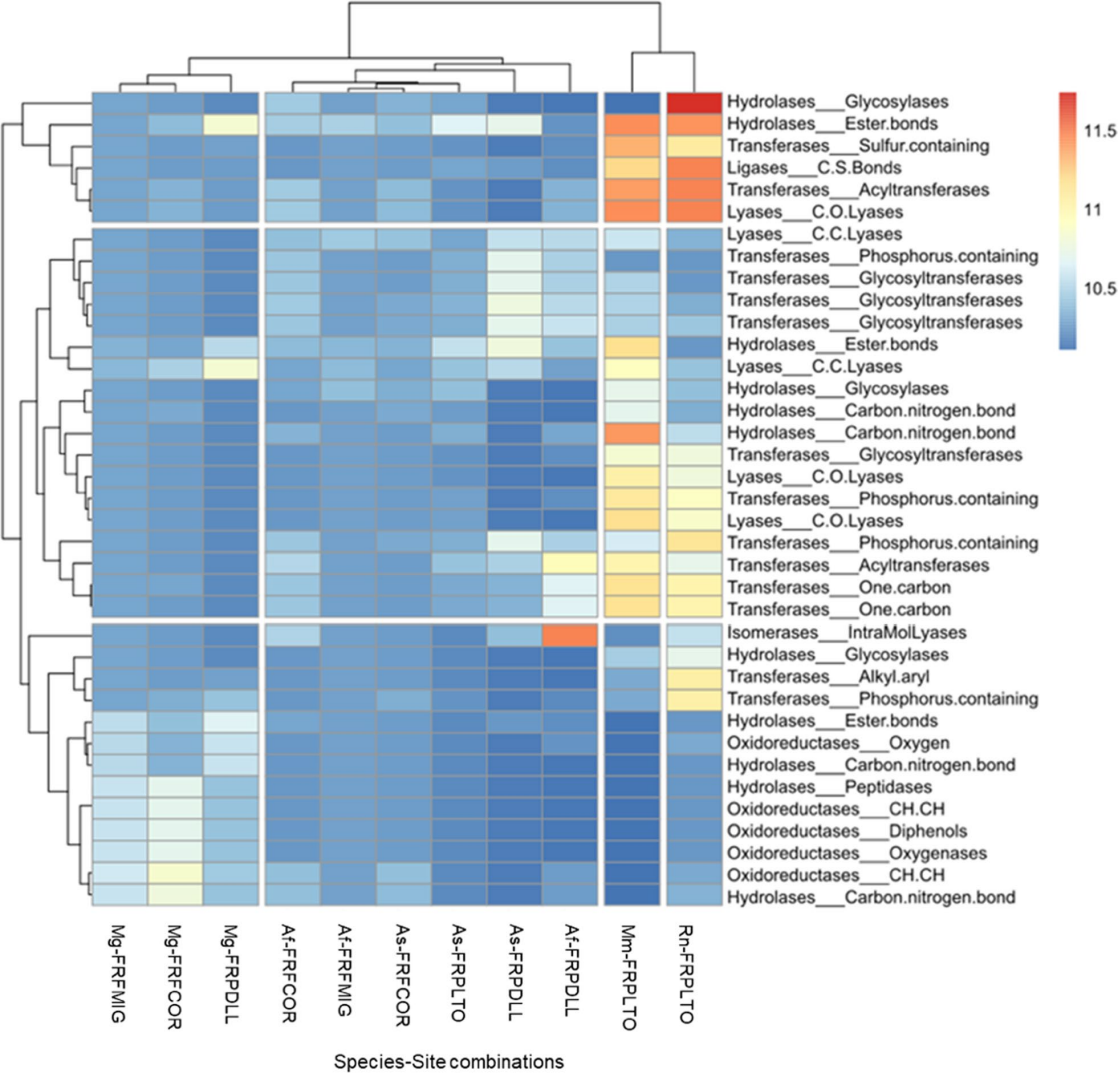
Heat map based on the DESeq2 results, considering GM functions (c-enzymes, EC) with significantly different abundances between species-site combinations. The color gradient corresponds to the magnitude of the differences. Red values indicate a strong score and blue values a weak score. Species-site combinations and enzyme classes are ordered according to their profile

The abundance of gut microbiota pathways and enzymes varied between sites (Fig. 1B, step4). We found 236 out of 383 metabolic pathways and 1064 out of 1969 enzymes with differential abundances between species-site combinations (Additional file 7: Table S3.4, Fig. 6; Additional file 12: Fig. S8B). The urban dweller species diverged from urban adapter species due to an over-representation of some hydrolases (ester bond group) and transferases (sulfur and phosphorus containing groups, acyltransferases, glycotransferases). Different enzyme classes showed differential abundance between sites for the three urban adapter species. Hydrolases (ester bond group) were more abundant in urbanized sites for *M. glareolus* and *A. sylvaticus*. For *M. glareolus*, we also detected an over-representation of lyases (C.C. group) and an under-representation of hydrolases (peptidases and carbon nitrogen group) and oxidoreductases in FRPDLL compared to FRFMIG and FRFCOR. For A. *flavicollis*, we found an over-representation of transferases (acyltransferases and one carbon) and isomerases (intramolecular lyases) in FRPDLL compared to FRFMIG and FRFCOR. The results for the taxonomic families and pathways are summarized in additional files (Additional file 7: Table S3.4, Additional file 12: Fig. S8A).

## Discussion

### Small mammal communities change with urbanization

Urbanization represents one of the most extreme forms of land use change [42]. It is associated with significant modifications of wildlife community composition, resulting in an overall decrease of diversity with only specific species capable of adapting to these highly anthropized environments [82]. The key pre-requisites for this ability to cope with urban environments include plasticity (ecological, behavioral), dispersal abilities, high reproductive output and a wide spectrum of habitat and diet requirements [82, 111]. Here we sampled small mammals in forested sites with different levels of urbanization, defined according to the extent of artificial features as well as the size and fragmentation of the forests. We observed marked changes in the composition of small mammal communities between sampling sites, that are likely to be mediated by urbanization, as previously shown in other studies [31, 86]. This enabled us to classify small mammal species into three categories with regard to urbanization, namely the urban avoiders, among which one endangered species in some part of France, the urban adapter and the urban dweller species.

The differences in small mammal community composition between sites may result from differences in the ecology and life-history traits of these animals, shaping their ability to adapt to the rapid changes associated with urbanization, and limiting biodiversity in more urbanized areas (e.g. habitat fragmentation and pollution, [31]). Besides, urban sites can offer abundant and continuous resources, as well as new refuges for small mammals, which can lead to the coexistence of adapter and urban dweller species [16, 71]. These processes may lead to intermediate levels of biodiversity in intermediate habitats, as proposed by the intermediate disturbance hypothesis (Connell 1978; [85]). The observed patterns of diversity and composition of small mammal communities in the peri-urban park FRDLL could corroborate this hypothesis, as already shown for other mammals (e.g. [30]).

### Host species-driven flexibility of the gut microbiota: the influence of categories of urbanization response

Our findings showed that species identity was a foremost determinant of GM diversity and composition. As noted by Kohl [49], the GM exhibited inter-individual variation but remains relatively conserved based on the identity of the host species. Such phylosymbiosis (observed concordance between the GM and the host species) has already been described in former studies on sympatric species of small mammals [48, 110].

Despite this undeniable role of host phylogeny in microbial structure, our results emphasized significant variations in the diversity and composition of the GM among different species categories, potentially reflecting their responses to urbanization. Specifically, we observed that the structure of gut microbiota differed between categories, but were similar within each category, whatever the phylogenetic distance between small mammal species. Varudkar & Ramakrishnan [102] previously reported a similar pattern while comparing the GM of two phylogenetically related rats in rural and urban habitats. Habitat emerged as the main predictor of GM diversity and composition. More studies are now required to corroborate the strong impact of responses to urbanization on inter-specific differences in GM. Specifically, experimental manipulation of gut microbiome composition (e.g. fecal microbiota transfer between individuals) would enable an assessment of the impact of these differences in GM between host species categories on critical traits for coping with urban environments (e.g. behavior, reproduction, diet). Additionally, it could shed light on the relationships between GM and host health, through GM influences on hosts’ sensibility to pathogens (e.g. [91, 99]).

### Stochasticity, a major process shaping microbial diversity and composition in urban dweller and avoider species

Our study highlighted the influence of distinct ecological processes on the GM diversity and composition of urban dweller vs adapter species. Urban dweller species had higher levels of intraspecific variance in their GM composition than adapter species. This may be due to larger ecological niche, for example with differences in diet between urban and rural areas, as has been reported in coyotes [95]. In rural forests, small mammals mainly eat seeds, fruits, and insects [116], whereas in urban areas, they may have access to a wider range of food sources such as garbage and pet food [32]. However, other factors beyond diet may also be at play, as this pattern of GM variance was observed even when species were found in sympatry and potentially sharing their resources and habitats.

Our results provided evidence for a strong influence of stochastic processes on the GM diversity and composition of urban dweller species. High variance of the GM in these species could thus reflect the deregulation of the bacterial composition and dysbiosis, with individuals acquiring different microbiota due to stochastic processes [117]. Although this phenomenon has already been observed in urban environments [93, 95], it remains important in the future to investigate how this stochasticity and high variance in GM composition impact the physiology, health and fitness of small mammal species. This is all the most important as large-scale GM studies conducted on humans have suggested that stochastic processes strongly influenced the microbiome composition, without any effects detected on host health (e.g. [26]). However, a recent meta-analysis has shown that over 27 microbiome associated studies conducted on humans, about half of them showed an impact of stochasticity on health disorders [60], therefore confirming the Anna Karenina Principle for some diseases [117].

In addition, we detected higher levels of functional richness and lower levels of functional redundancy in urban dwellers compared to adapter species. Dweller species had a GM that consisted of a small number of specialized taxa with unique functions. The GM diversity and composition might hence have contributed to the specialization of these dweller species to urban environments [64]. For instance, the urban dweller species showed a lower representation of Muribaculaceae and Rikenallaceae bacteria (as seen in mice fed with low fiber diet, e.g. [106]) and an over-representation of Helicobacteraceae, as previously found in mice fed at high-fat diet [3].

The higher representation of several enzymes and metabolic pathways in dwellers compared to urban adapter species suggest that these functions could play a critical role in urban dweller species adaptation in cities. Specifically, these functions could counteract the damaging anthropogenic pressures encountered in such disturbed environment, enabling urban dwellers to settle and persist while being "sick". As such, in humans, genes encoding for hydrolases are overexpressed in cities, and some of the metabolic functions related to these enzymes are involved in the regulation of cardiovascular diseases, inflammatory responses and neurological diseases [67].

The gut microbiota (GM) of the urban avoider species studied here, the soricomorph *Crocidura leucodon*, exhibited similar patterns (low alpha diversity, high inter-individual variance in GM composition) driven by the same processes (low levels of bacterial competition and functional redundancy, high rate of stochasticity) than other urban dweller species, in particular the phylogenetically close *C. russula*. This similarity may stem from ecological specialization rather than urban adaptation (e.g. [118]). The GM may play a role in the expansion or specialization of the host niche, known as the extended phenotype [69]*.* In this study, *C. leucodon* was trapped in a single forest. As it inhabits open woodland environments and agricultural areas, it would be interesting to investigate the ability of this urban avoider species to resist or respond to rapid environmental changes, as those experienced due to climatic and anthropogenic changes.

### Variable influence of ecological processes in shaping the gut microbiota of urban adapter species in response to urbanization

We next focused on urban adapter species and investigated GM flexibility between sites, whose environmental differences mainly reflected urbanization. We revealed an impact of urbanization on GM diversity and composition, as previously described for other mammals (e.g. [94, 95]).

Higher values of GM diversity and marked differences in GM composition were observed in the peri-urban park FRPDLL compared to forests in the three adapter species. The patterns for taxonomic richness were less pronounced when considering the Shannon index, meaning that changes in bacterial diversity were mainly driven by the presence of rare species. This could suggest a potential cascading effect of changes in host communities on host microbial communities, or a similar impact of environmental disturbance on both communities with higher species diversity maintained in environments with intermediate disturbance. This intermediate disturbance hypothesis (Connell, 1978; [115]) could explain that microbial diversity in peri-urban parks (e.g. FRPDLL) originates from both urban and forest ecosystems [85], a pattern that has been detected in several empirical studies. With regard to GM, the higher diversity in these habitats with intermediate levels of urbanization could be mediated by a larger diversity of food available (e.g. [36]) resulting in larger ecological niche and greater external microbial diversity [110].

Besides, we provided evidence for higher levels of functional redundancy in urban parks than in forests. We have detected signals of phylogenetic clustering resulting from strong bacterial filtering [109]. When a species of bacteria cannot fulfill a specific function due to unfavorable environmental conditions, another species or group can step in. This ensures the stability of essential functions and the robustness of GM [50, 68]. In urban environments where wildlife is submitted to rapid and negative perturbations, functional redundancy can enable buffering of these disturbances by mitigating functional loss and securing important metabolic interaction networks, consequently conferring resilience [35].

However, if this functional redundancy becomes excessive, particularly due to intense environmental selection, it could lead to a homogenization of functions, making the microbiome less resilient to unexpected environmental changes. This urban homogenization has been observed in urban microbial soil [19]. Lastly, the relative importance of selection in shaping the GM of these urban adapter species was slightly higher in rural than in urban forests and we detected changes in enzymes and metabolic pathways between sites that could be shared by several adapter species. Altogether, these results indicated that the GM composition of urban adapter species are spatially stable despite contrasted levels of urbanization. Besides, some variability in the observed patterns emerged when comparing the three urban-adapter species investigated in this study. Notably, the changes in GM diversity and composition observed in *A.* *sylvaticus* between sites were less pronounced than in *M. glareolus* and *A. flavicollis*. It is likely that species differ in their abilities to cope with urbanization, even within a given category, owing to variations in key life history traits such as behavior, diet, reproduction. *A. sylvaticus* was the only adapter species established in FRPLTO, which suggests better abilities to respond to urbanization. However, current small mammal databases referencing life history traits have not enabled the identification of key features associated with this capacity to cope with urbanization (eg Pantheria, Elton, Additional file 6: Table S2). This is likely due to the paucity and incompleteness of these databases: the available data correspond to few locations, whereas the geographic distribution of these species may be large, with spatial variability in life-history traits).

Surprisingly, we found no evidence of signature of dysbiosis in urban sites for these urban adapter species, contrary to what could have been expected from other studies (e.g. for the spiny rat *Proechimys semispinosus* [25]). This suggests GM resistance or resilience [68, 90] in the face of such environmental changes [1, 64, 104]), with the impact of stochasticity being dampened by functional redundancy [68, 90]. Temporal surveys are now necessary to corroborate this effect of urbanization on GM dynamics and evolution in urban adapter species and to better understand how these responses to urbanization may affect their fitness.

## Conclusions

This study corroborated the strong interlinkages existing between small mammal communities and their GM, that might change with hosts’ and abiotic features. As such, feedback loops might occur between host communities and their microbiota in response to environmental perturbations [63, 66]. The gut microbiome is partly inherited and shaped by the external environment. As such, it may react to shifts in host communities that resulted from environmental disturbances, through changes in microbiota availability [14]. But the GM's plasticity can provide the hosts with the capacity to acclimate / adapt to rapid environmental perturbations. This plasticity may favor particular individuals or species through its impacts on host health (immunity, feeding behaviour, …), what may in turn cascade into modifications in the assemblage of host communities (Albery et al., 2016). These feedback loops are therefore dynamic processes where environmental disturbances may affect host behavior or diet, with impacts on the composition and diversity of the GM potentially leading to dysbiosis and maladaptation. Conversely, alterations in the microbiota due to abiotic conditions (e.g. pollution, temperature…) can influence the host's ability to adapt to its ecological niche.

In our study, urbanization seemed to impact the diversity and composition of GM, either as a cause or a consequence of the host species’ ability to cope with environmental changes. The observation of higher levels of Helicobacteraceae and hydrolases (ester bond groups) in dweller species, or in adapter species when trapped in urban parks, raises the question of the ecological advantage of these taxa and enzymes when coping with urbanization. Notably, understanding the links between these changes in GM and variations in diet between habitats of contrasted urbanization levels would provide valuable insights into the role of GM in the ecological acclimatation/adaptation of small mammals in urban environments.

In a more general perspective, the patterns detected in this study now deserve further investigations to deepen our understanding of the impacts of GM shifts between individuals, sites and species, on host health. Previous works have highlighted associations between GM diversity and/or composition on small mammal sensibility to pathogens (e.g. [9]). Further research should now be conducted to assess the impact of urbanization on the zoonotic risks associated with small mammals, considering not only changes in pathogens communities in urban environments, but also the complex interactions between small mammals communities, their GM and the pathogens. This is particularly crucial in developed countries where the promotion of green cities is emphasized, as well as in developing countries undergoing dramatic urban expansion.

### Supplementary Information

**Additional file 1. Fig. S1.** Spatial information about the sampling sites.

**Additional file 2. Table S1.** Small mammal communities analyses.

**Additional file 3.** Outliers.

** Additional file 4. Fig. S2.** Phylogeny and communities composition of small mammal species.

**Additional file 5. Fig. S3.** Barplot of relative abundance of gut microbiota at phylum and family levels.

**Additional file 6. Table S2.** Alpha diversity metrics and statistics for the gut microbiome.

**Additional file 7. Table S3.** Beta diversity metrics and statistics for the gut microbiome.

**Additional file 8. Fig.  S4.** Distance-based redundancy analysis (db-RDA) based on the composition of the gut microbiota of small mammal species.

**Additional file 9. Fig. S5.** Tanglegrams.

**Additional file 10. Fig. S6.** Boxplot of the distance to the centroid of each individual within the group of sites.

**Additional file 11. Fig. S7.** Processes responsible for bacterial assembly and turnover in each combination of small mammal species and site.

**Additional file 12. Fig. S8.** Heat map based on DESeq2 results illustrating significantly different abundances between species-site combinations, considering gut microbiota.

## Data Availability

Raw data and scripts are available on zenodo repository (https://zenodo.org/record/8143272).
