# Supplementary material for: Exploring the potential effects of forest urbanization on the interplay between small mammal communities and their gut microbiota
Source: Anim Microbiome. 2024 Mar 25;6:16. doi: 10.1186/s42523-024-00301-y (PMC10964555; doi:10.1186/s42523-024-00301-y)
Supplement: Supplementary file 1 — Additional file 1. Fig. S1. Spatial information about the sampling sites. [file 42523_2024_301_MOESM1_ESM.docx]

Exploring the potential effects of forest urbanization on the interplay between small mammal communities and their gut microbiota

Marie Bouilloud^a*^, Maxime Galanb, Julien Pradel^b^, Anne Loiseau^b^, Julien Ferrero^b^, Romain Gallet^b^, Benjamin Roche^c^, Nathalie Charbonnel^b^

**^a^** CBGP, IRD, CIRAD, INRAE, Institut Agro, Univ Montpellier, Montpellier, France

**^b^** CBGP, INRAE, IRD, CIRAD, Institut Agro, Univ Montpellier, Montpellier, France

**^c^** MIVEGEC, IRD, CNRS, Univ Montpellier, Montpellier, France

***Corresponding author at: Centre de Biologie pour la Gestion des Populations, 750 avenue agropolis, 34988 Montferrier sur Lez, France.**

***Email address:*** marie.bouilloud@gmail.com (M. Bouilloud).

Supplementary Figure 1


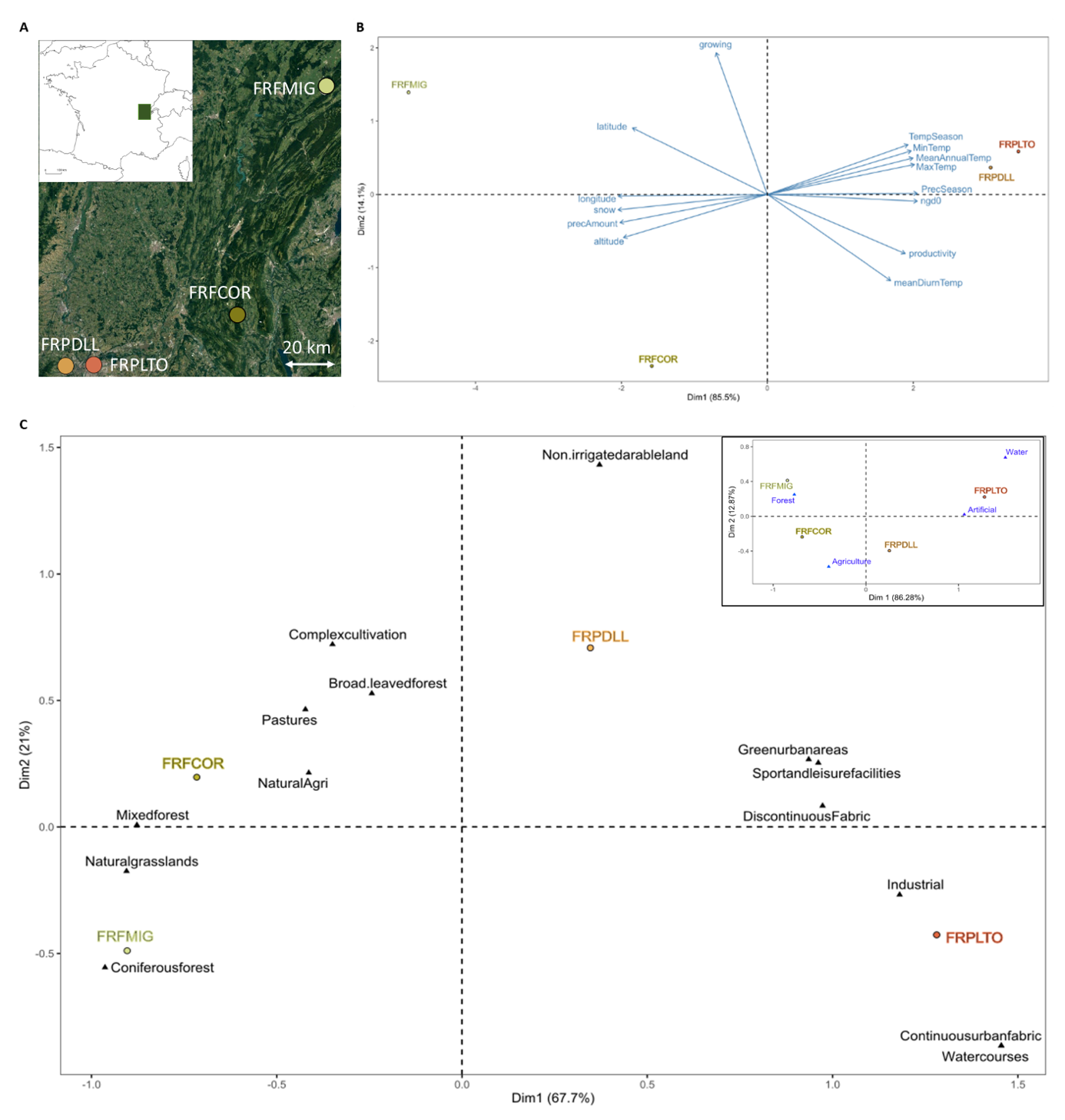


**Fig. S1.** Spatial information about the sampling sites. A) Map of the sampling sites, indicating the location of the sites at the national and local scales. B) Principal component analysis of sampling sites based on biogeoclimatic indices (Chelsa database) and geographical factors (latitude, longitude and altitude). C) Canonical analysis of the proportion of land use (triangle) at each site (coloured dots). The data were extracted from the Corine Land Cover database (level of accuracy : 3), using a buffer of 3000 m around the barycentre of each site. The figure on the top right of this graph represents the results of the same analysis performed using information of Corine Land Cover extracted for a level of accuracy of 1.
