# Supplementary material for: Exploring the potential effects of forest urbanization on the interplay between small mammal communities and their gut microbiota
Source: Anim Microbiome. 2024 Mar 25;6:16. doi: 10.1186/s42523-024-00301-y (PMC10964555; doi:10.1186/s42523-024-00301-y)
Supplement: Supplementary file 3 — Additional file 3. Outliers. [file 42523_2024_301_MOESM3_ESM.docx]

# Exploring the effects of forest urbanization on the interplay between small mammal communities and their gut microbiota

Marie Bouilloud^a*^, Maxime Galanb, Julien Pradel^b^, Anne Loiseau^b^, Julien Ferrero^b^, Romain Gallet^b^, Benjamin Roche^c^, Nathalie Charbonnel^b^

**^a^** CBGP, IRD, CIRAD, INRAE, Institut Agro, Univ Montpellier, Montpellier, France

**^b^** CBGP, INRAE, IRD, CIRAD, Institut Agro, Univ Montpellier, Montpellier, France

**^c^** MIVEGEC, IRD, CNRS, Univ Montpellier, Montpellier, France

***Corresponding author at: Centre de Biologie pour la Gestion des Populations, 750 avenue agropolis, 34988 Montferrier sur Lez, France.**

***Email address :*** [marie.bouilloud@gmail.com](mailto:marie.bouilloud@gmail.com) (M. Bouilloud).

# Supplementary File

Description: Analyses of the differences between the gut microbiota of small mammals found dead or alive in traps


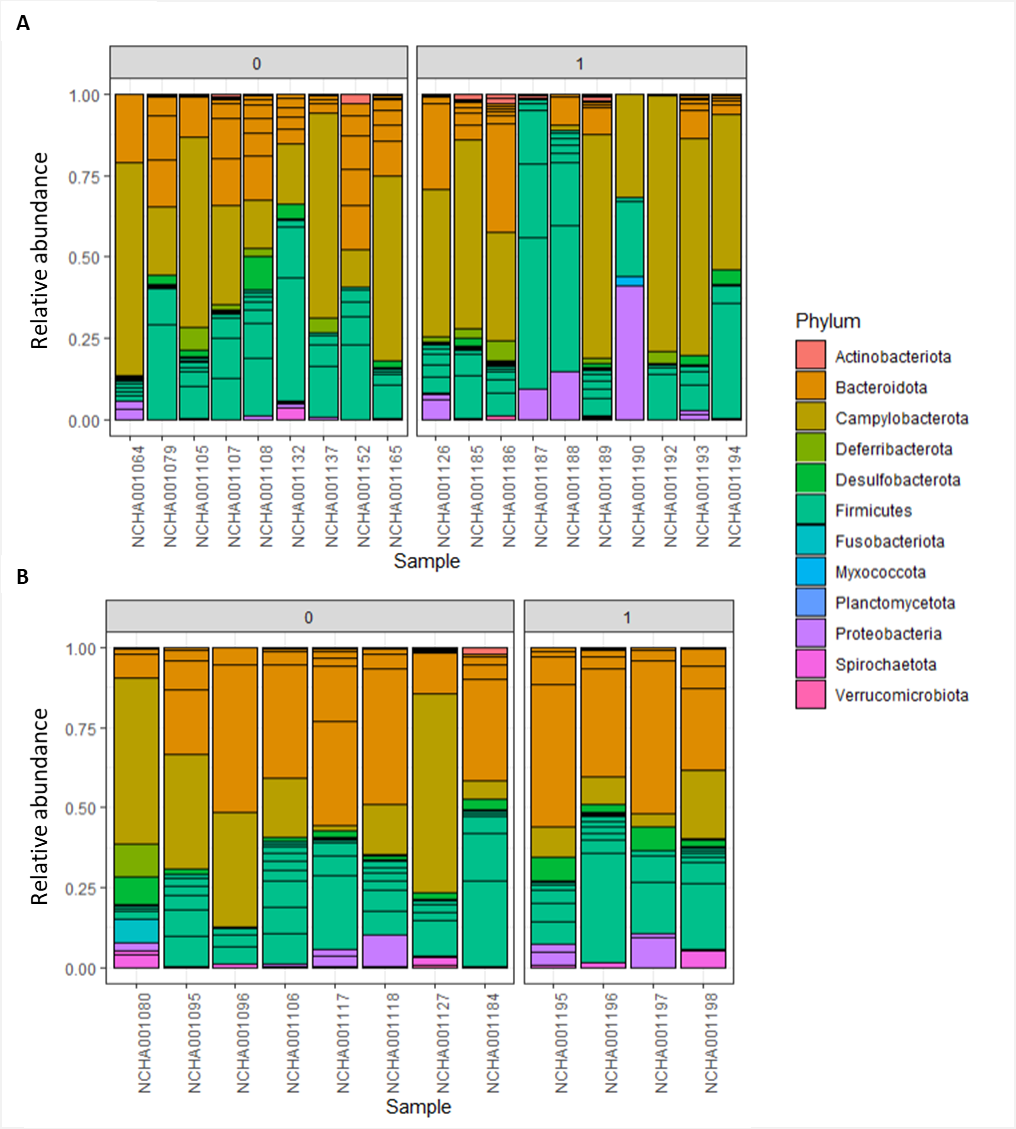


**Supp. File 1**. Differences in the relative abundance of ASVs at the phylum level between small mammals according to their status (code 0 = found alive in the trap, code 1= found dead in the trap) for A) *Mus musculus* and B) *Rattus norvegicus*. Each color corresponds to a phylum.


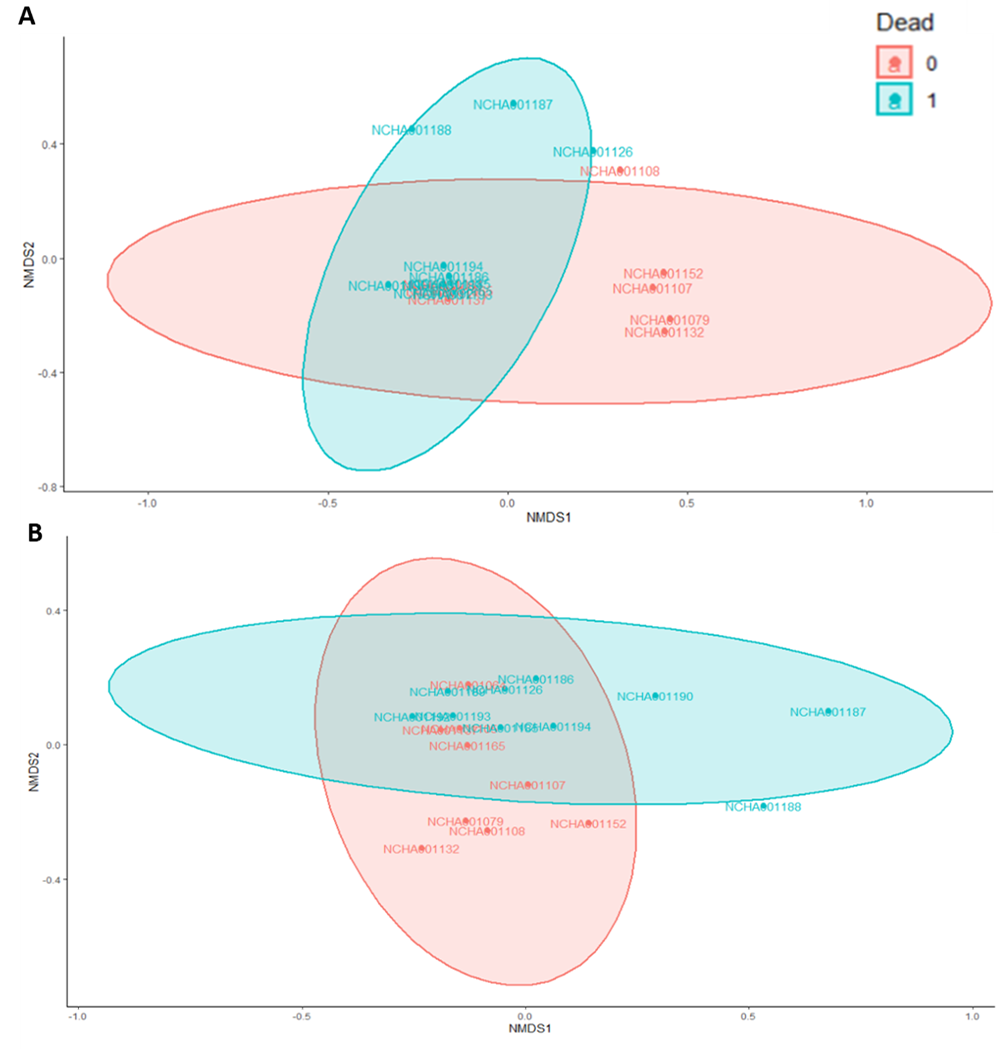


**Supp. File 2**. Differences in the composition of the GM (ASVs) at the phylum level according to small mammal status (code 0 = found alive in the trap, code 1= found dead in the trap) for A) *Mus musculus* and B) *Rattus norvegicus*

**Supp. File 3.** Statistical analysis (Adonis test) of the difference of GM composition between small mammals found dead or alive in the traps, for A) *Mus musculus* and B) *Rattus norvegicus*.

1. *Mus musculus*

1. *Rattus norvegicus*


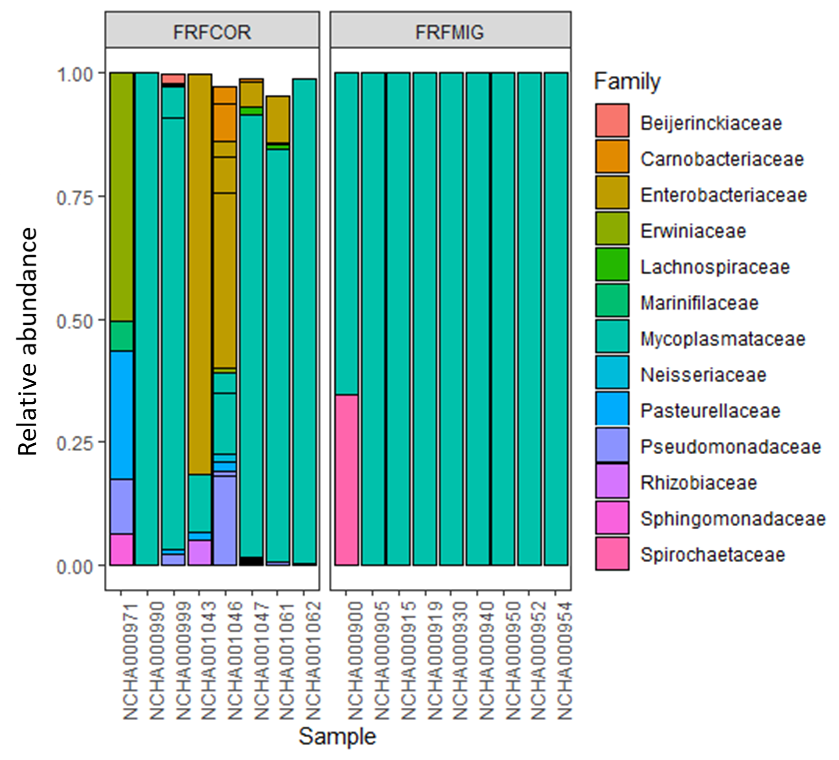


**Supp. File 4.** Relative abundance of GM taxa at the family level in *Glis glis* (each color corresponding to one family). We can observe an overabundance of the family *Mycoplasmataceae*, only few individuals had other taxa.
