# Supplementary material for: Exploring the potential effects of forest urbanization on the interplay between small mammal communities and their gut microbiota
Source: Anim Microbiome. 2024 Mar 25;6:16. doi: 10.1186/s42523-024-00301-y (PMC10964555; doi:10.1186/s42523-024-00301-y)
Supplement: Supplementary file 4 — Additional file 4. Fig. S2. Phylogeny and communities composition of small mammal species. [file 42523_2024_301_MOESM4_ESM.docx]

Exploring the effects of forest urbanization on the interplay between small mammal communities and their gut microbiota

Marie Bouilloud^a*^, Maxime Galanb, Julien Pradel^b^, Anne Loiseau^b^, Julien Ferrero^b^, Romain Gallet^b^, Benjamin Roche^c^, Nathalie Charbonnel^b^

**^a^** CBGP, IRD, CIRAD, INRAE, Institut Agro, Univ Montpellier, Montpellier, France

**^b^** CBGP, INRAE, IRD, CIRAD, Institut Agro, Univ Montpellier, Montpellier, France

**^c^** MIVEGEC, IRD, CNRS, Univ Montpellier, Montpellier, France

***Corresponding author at: Centre de Biologie pour la Gestion des Populations, 750 avenue agropolis, 34988 Montferrier sur Lez, France.**

***Email address:*** marie.bouilloud@gmail.com (M. Bouilloud).

Supplementary Figure 2

**
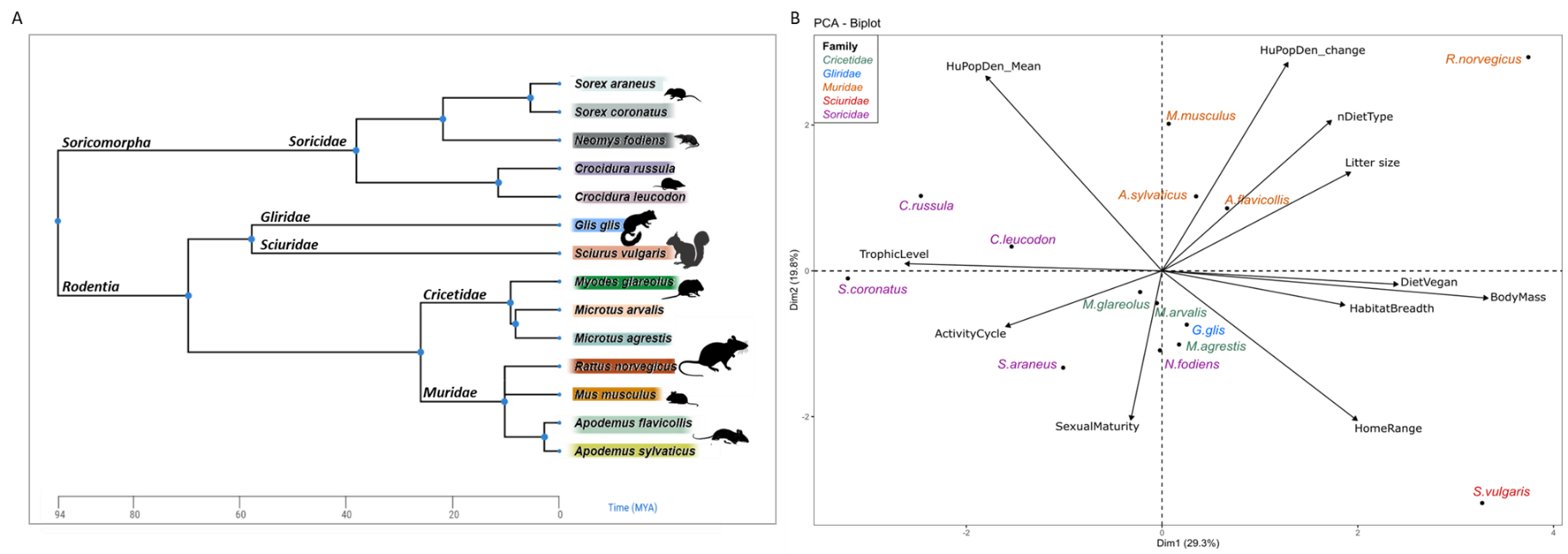
Fig. S2.1.** Phylogeny of small mammals. Phylogenetic tree of small mammal species trapped in this study, built from *TimeTree of Life 5* (Kumar et al., 2022). The taxonomic ranks (order, family, genus and species) have been annotated on the tree. Nodes correspond to hypothetical common ancestors. Colors and silhouettes illustrate genus or families.

#
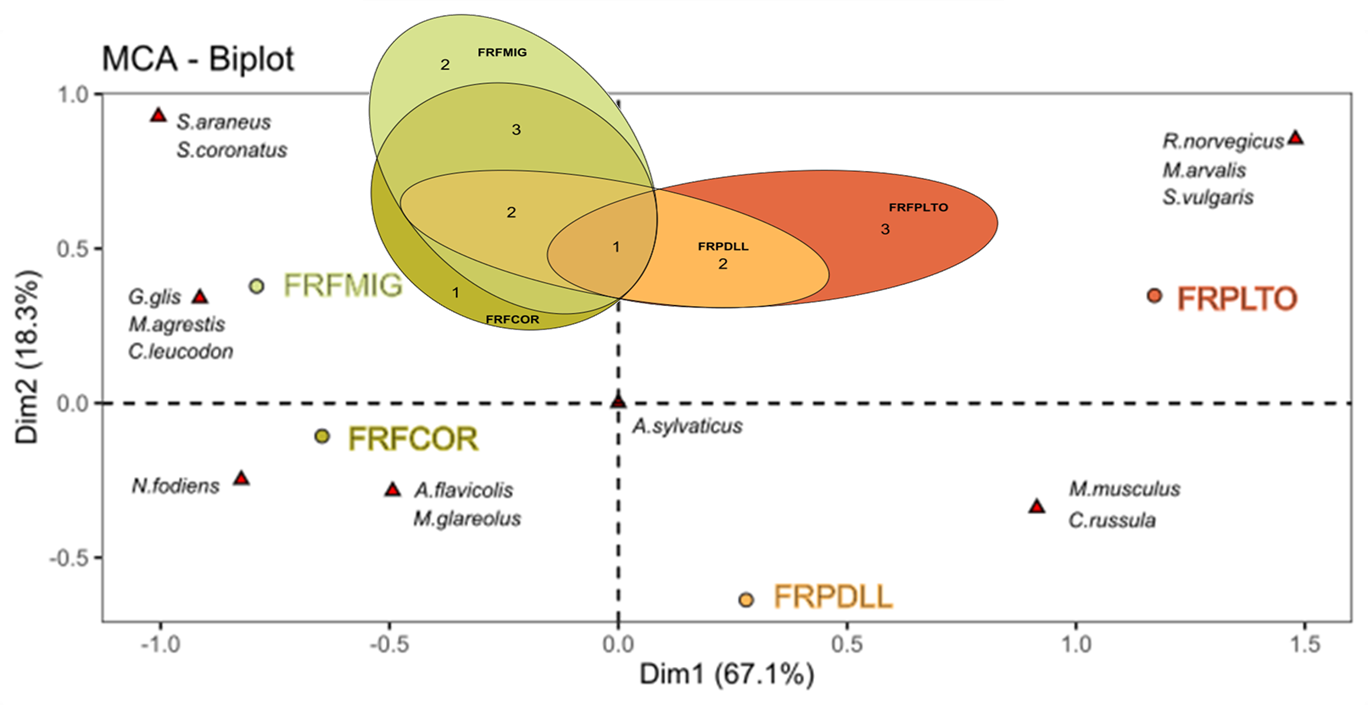


**Fig. S2**.**2** Multiple correspondence analysis (MCA) of small mammal species (represented by a red triangle) by sites (colored dots, FRFMIG, FRFCOR, FRPDLL and FRPLTO). The Euler diagram shows more precisely the number of species shared between sites.
