# Supplementary material for: Exploring the potential effects of forest urbanization on the interplay between small mammal communities and their gut microbiota
Source: Anim Microbiome. 2024 Mar 25;6:16. doi: 10.1186/s42523-024-00301-y (PMC10964555; doi:10.1186/s42523-024-00301-y)
Supplement: Supplementary file 5 — Additional file 5. Fig. S3. Barplot of relative abundance of gut microbiota at phylum and family levels. [file 42523_2024_301_MOESM5_ESM.docx]

Exploring the effects of forest urbanization on the interplay between small mammal communities and their gut microbiota

Marie Bouilloud^a*^, Maxime Galanb, Julien Pradel^b^, Anne Loiseau^b^, Julien Ferrero^b^, Romain Gallet^b^, Benjamin Roche^c^, Nathalie Charbonnel^b^

**^a^** CBGP, IRD, CIRAD, INRAE, Institut Agro, Univ Montpellier, Montpellier, France

**^b^** CBGP, INRAE, IRD, CIRAD, Institut Agro, Univ Montpellier, Montpellier, France

**^c^** MIVEGEC, IRD, CNRS, Univ Montpellier, Montpellier, France

***Corresponding author at: Centre de Biologie pour la Gestion des Populations, 750 avenue agropolis, 34988 Montferrier sur Lez, France.**

***Email address:*** marie.bouilloud@gmail.com (M. Bouilloud).

Supplementary Figure 3


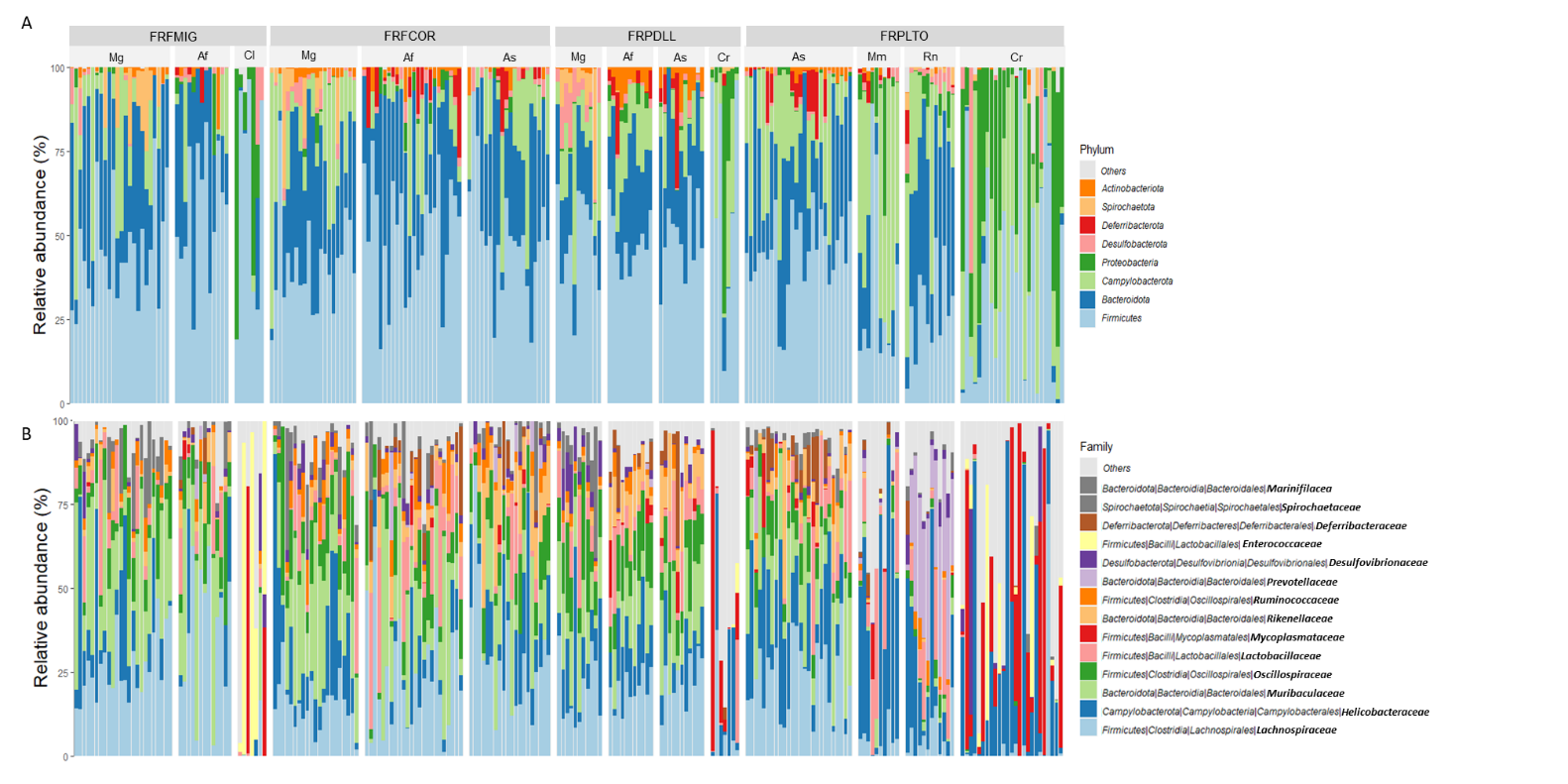


C

| **Sites** | **FRFMIG** | | | **FRFCOR** | | | **FRPDLL** | | | | **FRPLTO** | | | |
| --- | --- | --- | --- | --- | --- | --- | --- | --- | --- | --- | --- | --- | --- | --- |
| **Host species** | Mg | Af | Cl | Mg | Af | As | Mg | Af | As | Cr | As | Mm | Rn | Cr |
| **Number of individuals** | 24 | 13 | 7 | 21 | 24 | 20 | 11 | 11 | 11 | 7 | 26 | 10 | 12 | 25 |

**Fig. S3.** Bar plot of the relative abundance (%) of A) Phylum and B) Family of small mammal gut microbiota, for each “species-site” combination. The Family legends correspond to Kingdom/Phylum/Class/Order/Family. C) Summary of the number of individuals per site (FRFMIG, FRFCOR, FRPDLL, FRPLTO) and species (*Myodes glareolus* Mg, *Apodemus flavicollis* Af, *Crocidura* *leucodon* Cl, *Apodemus sylvaticus* As, *Crocidura russula* Cr, *Mus musculus* Mm, *Rattus norvegicus* Rn) included in all gut microbiome analyses.
