# Supplementary material for: Exploring the potential effects of forest urbanization on the interplay between small mammal communities and their gut microbiota
Source: Anim Microbiome. 2024 Mar 25;6:16. doi: 10.1186/s42523-024-00301-y (PMC10964555; doi:10.1186/s42523-024-00301-y)
Supplement: Supplementary file 9 — Additional file 9. Fig. S5. Tanglegrams. [file 42523_2024_301_MOESM9_ESM.docx]

Exploring the effects of forest urbanization on the interplay between small mammal communities and their gut microbiota

Marie Bouilloud^a*^, Maxime Galanb, Julien Pradel^b^, Anne Loiseau^b^, Julien Ferrero^b^, Romain Gallet^b^, Benjamin Roche^c^, Nathalie Charbonnel^b^

**^a^** CBGP, IRD, CIRAD, INRAE, Institut Agro, Univ Montpellier, Montpellier, France

**^b^** CBGP, INRAE, IRD, CIRAD, Institut Agro, Univ Montpellier, Montpellier, France

**^c^** MIVEGEC, IRD, CNRS, Univ Montpellier, Montpellier, France

***Corresponding author at: Centre de Biologie pour la Gestion des Populations, 750 avenue agropolis, 34988 Montferrier sur Lez, France.**

***Email address:*** marie.bouilloud@gmail.com (M. Bouilloud).

Supplementary Figure 5


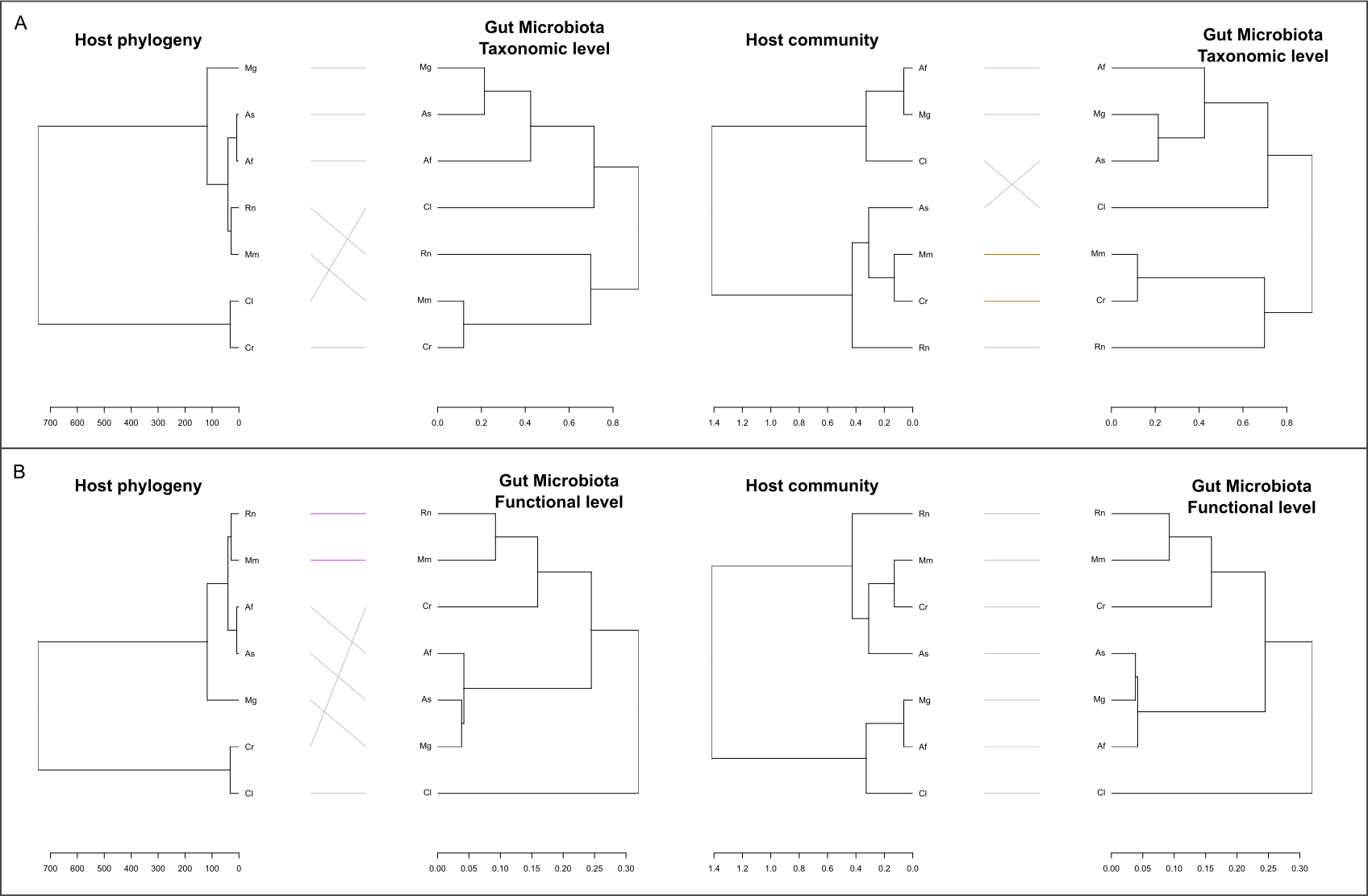


**Fig. S5** Tanglegrams showing concordance between 1) phylogenetic distance among small mammal and dissimilarities in function of their gut microbiota composition and 2) dissimilarities of niche for small mammal and dissimilarities in function of their gut microbiota composition. Concordance based on A) Taxonomy level (Weighted unifrac distance) and B) Functional level (Bray Curtis distance). The letters in tree correspond to species (Rn = R. *norvegicus*; Mm=*M. musculus*; As=*A. sylvaticus*; Af=*A. flavicollis*; Cr=*C. russula*; Cl=*C. leucodon*; Mg= *M. glareolus*)
