# Supplementary material for: Exploring the potential effects of forest urbanization on the interplay between small mammal communities and their gut microbiota
Source: Anim Microbiome. 2024 Mar 25;6:16. doi: 10.1186/s42523-024-00301-y (PMC10964555; doi:10.1186/s42523-024-00301-y)
Supplement: Supplementary file 11 — Additional file 11. Fig. S7. Processes responsible for bacterial assembly and turnover in each combination of small mammal species and site. [file 42523_2024_301_MOESM11_ESM.docx]

Exploring the effects of forest urbanization on the interplay between small mammal communities and their gut microbiota

Marie Bouilloud^a*^, Maxime Galanb, Julien Pradel^b^, Anne Loiseau^b^, Julien Ferrero^b^, Romain Gallet^b^, Benjamin Roche^c^, Nathalie Charbonnel^b^

**^a^** CBGP, IRD, CIRAD, INRAE, Institut Agro, Univ Montpellier, Montpellier, France

**^b^** CBGP, INRAE, IRD, CIRAD, Institut Agro, Univ Montpellier, Montpellier, France

**^c^** MIVEGEC, IRD, CNRS, Univ Montpellier, Montpellier, France

***Corresponding author at: Centre de Biologie pour la Gestion des Populations, 750 avenue agropolis, 34988 Montferrier sur Lez, France.**

***Email address:*** marie.bouilloud@gmail.com (M. Bouilloud).

Supplementary Figure 7


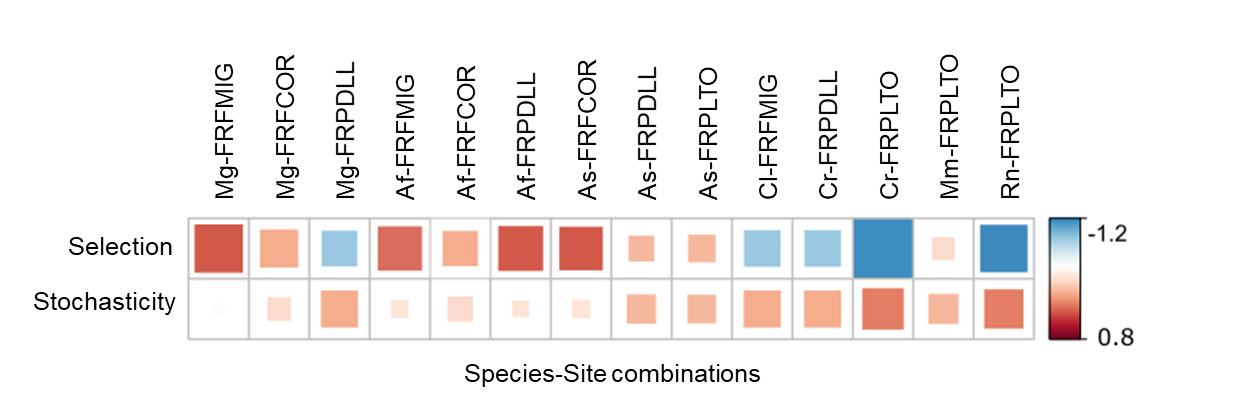


**Fig. S7.** Processes responsible for bacterial assembly and turnover in each combination of small mammal species and site. Corrplot representing the residual results of the chi-test analysis. The color gradient corresponds to the differences between observed and expected values. Negative values (blue) mean that processes are less observed than expected. Positive values (red) indicate that the share of processes is greater than expected.
