# Supplementary material for: Exploring the potential effects of forest urbanization on the interplay between small mammal communities and their gut microbiota
Source: Anim Microbiome. 2024 Mar 25;6:16. doi: 10.1186/s42523-024-00301-y (PMC10964555; doi:10.1186/s42523-024-00301-y)

Exploring the effects of forest urbanization on the interplay between small mammal communities and their gut microbiota

Marie Bouilloud^a*^, Maxime Galanb, Julien Pradel^b^, Anne Loiseau^b^, Julien Ferrero^b^, Romain Gallet^b^, Benjamin Roche^c^, Nathalie Charbonnel^b^

**^a^** CBGP, IRD, CIRAD, INRAE, Institut Agro, Univ Montpellier, Montpellier, France

**^b^** CBGP, INRAE, IRD, CIRAD, Institut Agro, Univ Montpellier, Montpellier, France

**^c^** MIVEGEC, IRD, CNRS, Univ Montpellier, Montpellier, France

***Corresponding author at: Centre de Biologie pour la Gestion des Populations, 750 avenue agropolis, 34988 Montferrier sur Lez, France.**

***Email address:*** marie.bouilloud@gmail.com (M. Bouilloud).

Supplementary Figure 8

**Fig. S8.** Heat map based on DESeq2 results illustrating significantly different abundances between species-site combinations, considering gut microbiota A) taxa (family level), B) metabolic pathways. The color gradient corresponds to the magnitude of the differences. Red values indicate a strong score and blue values a weak score. Species-site combinations and enzyme classes are ordered according to their profile.


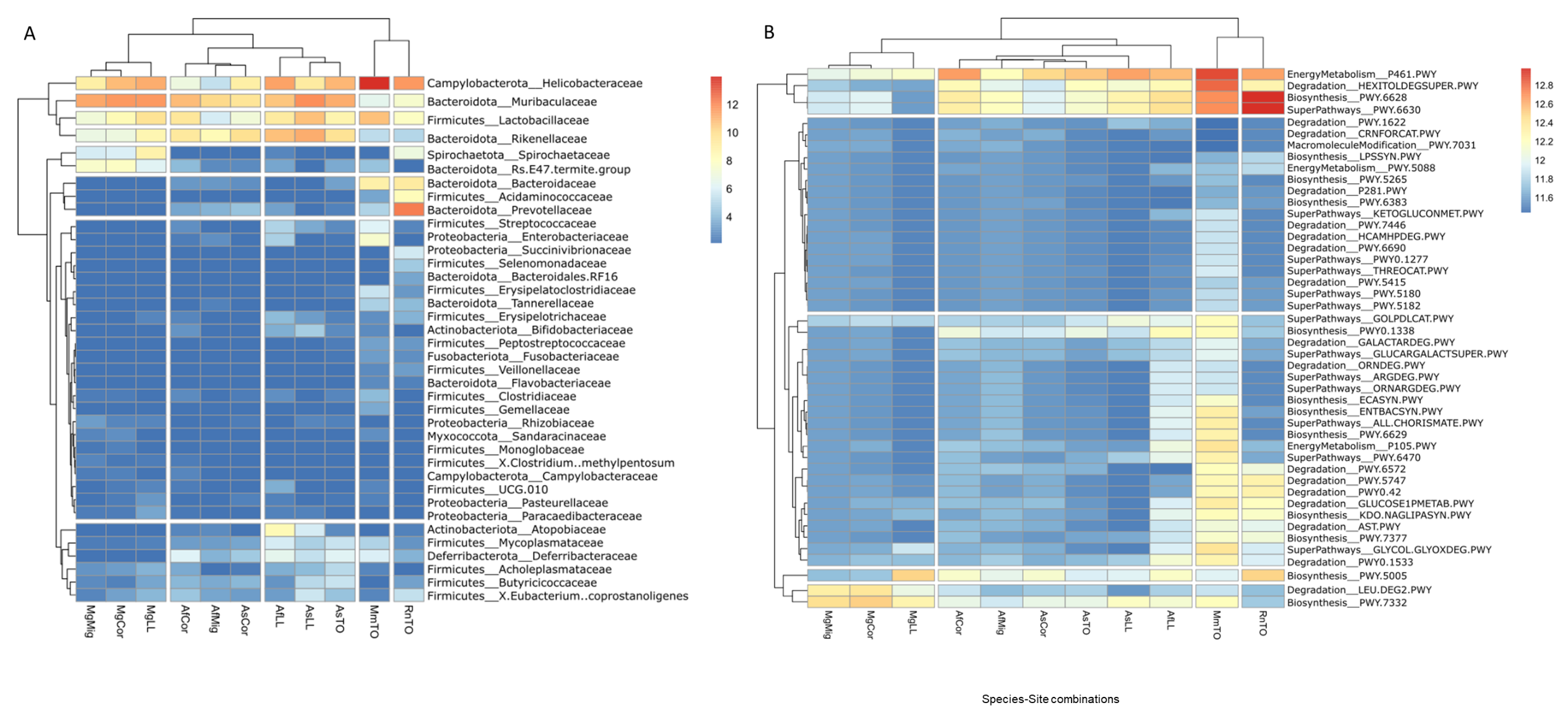

Supplement: Supplementary file 12 — Additional file 12. Fig. S8. Heat map based on DESeq2 results illustrating significantly different abundances between species-site combinations, considering gut microbiota. [file 42523_2024_301_MOESM12_ESM.docx]
